# Structural, Sorption, and Regeneration Properties of Poly(methacrylic acid): Poly(4-vinylpyridine) Interpolymer Systems for the Recovery of Rhenium and Molybdenum

**DOI:** 10.3390/polym17223054

**Published:** 2025-11-18

**Authors:** Arman Baishibekov, Dametken Fischer, Talkybek Jumadilov, Saniya Temirova, Sultan Yulusov, Bagdat Altaibayev, Diana Karim

**Affiliations:** 1 Institute of Metallurgy and Ore Beneficiation JSC, Satbayev University, Shevchenko Str., 29/133, Almaty 050010, Kazakhstan; a.baishibekov@satbayev.university (A.B.); s.temirova@satbayev.university (S.T.); s.yulussov@satbayev.university (S.Y.); b.altaibayev@satbayev.university (B.A.); d.karim@satbayev.university (D.K.); 2 A.B. Bekturov Institute of Chemical Sciences JSC, Satbayev University, Sh. Ualikhanov Str., 106, Almaty 050010, Kazakhstan; jumadilov@mail.ru

**Keywords:** rhenium, molybdenum, interpolymer system, poly(methacrylic acid) (PMAA), poly(4-vinylpyridine) (P4VP), sorption, desorption, FTIRspectroscopy, thermoanalysis, selective extraction, hydrometallurgical solutions

## Abstract

The sorption, structural, and thermal properties of the interpolymer system based on polymethacrylic acid (PMAA) and poly-4-vinylpyridine (P4VP) were studied at different component ratios for the extraction of rhenium (ReO_4_^−^) and molybdenum (${MoO}_{4}^{2-}$) ions from model solutions. The results showed that sorption efficiency depends on both composition and contact time. Systems with molar ratios of 3:3 and 2:4 exhibited the highest activity, reaching more than 90% extraction for rhenium and up to 94% for molybdenum after 48 h. FTIR spectra indicated the involvement of carboxyl and pyridine groups in complex formation with the metal ions, as evidenced by band shifts and the appearance of new absorption features. Thermogravimetric analysis (TGA) and differential scanning calorimetry (DSC) demonstrated that modification of PMAA with P4VP enhances the thermal stability of the system, which is maintained even after desorption. The system with the optimal composition showed structural stability and the ability to regenerate without a notable decrease in sorption capacity. Sorption performance was preserved across a wide pH interval, with maximum values at pH 2–4, which makes the system suitable for hydrometallurgical applications. Comparison with literature data indicates agreement with current approaches to rare-metal recovery, while the PMAA–P4VP system is distinguished by improved stability and potential for further development. These findings provide a basis for the design of selective and environmentally safe processes for rhenium and molybdenum recovery, relevant to the conditions of the Republic of Kazakhstan, where technogenic solutions rich in these metals are available.

## 1. Introduction

Rhenium and molybdenum belong to the class of dispersed elements whose exceptional physicochemical properties determine their broad application in high-temperature alloys, catalysis, and microelectronics [1,2,3,4,5]. Despite their technological significance, these metals are rarely found in nature and require complex methods for extraction. Rhenium is mainly a trace component of copper–molybdenum ores, while molybdenum occurs in sulfide and oxide forms [6,7,8]. Owing to the scarcity of natural deposits and the increasing industrial demand, attention has shifted toward the recovery of these elements from secondary and technogenic sources, including spent catalysts and metallurgical residues [4,8]. For Kazakhstan, where such sources are abundant, the development of selective, recyclable sorbents has both scientific and practical relevance [6,7].

Conventional extraction methods—pyrometallurgical, solvent extraction, and precipitation—require aggressive reagents and high energy consumption, generating large volumes of waste [9,10,11,12,13,14]. In contrast, polymeric and interpolymer sorbents offer mild operating conditions, tunable selectivity, and structural stability [15,16,17,18,19,20,21,22,23,24,25,26,27,28]. Their technical efficiency and limitations are summarized in Table A1 (Appendix A). Among them, interpolymer systems (IPS) formed by weak polyacids and polybases exhibit unique cooperative effects: mutual activation alters the degree of ionization, modifies polymer conformation, and creates new adsorption centers capable of donor–acceptor interactions with metal oxyanions [28,29,30,31,32]. Despite the progress achieved, the mechanisms governing the selective binding of rhenium and molybdenum in such systems remain insufficiently clarified. Most studies have examined either Re(VII) or Mo(VI) separately [8,28,31], neglecting the competitive sorption and the relationship between interpolymer composition, structure, and kinetics. Furthermore, few works have compared equilibrium models for describing oxyanion sorption on heterogeneous polymer matrices.

The present work focuses on an interpolymer system based on poly(methacrylic acid) (PMAA) and poly(4-vinylpyridine) (P4VP), which provides complementary acidic (–COOH) and basic (–C_5_H_4_N) functional groups. This combination enables simultaneous electrostatic and coordination interactions with Re(VII) and Mo(VI) oxyanions [33,34]. Through comprehensive sorption studies supported by FTIR and thermal analyses (TGA/DSC), the influence of polymer composition, pH, and contact time on ion uptake is established. Nonlinear equilibrium and kinetic modeling identify the most appropriate adsorption model and elucidate the interaction mechanism. The results highlight the novelty and importance of the PMAA/P4VP system as a regenerable and selective adsorbent for the environmentally sustainable recovery of rhenium and molybdenum. The sorption of rhenium Re(VII) and molybdenum Mo(VI) from aqueous solutions is a critical process for hydrometallurgical applications. The PMAA–P4VP interpolymer system has demonstrated effective sorption and selective recovery of these metals. This study investigates its sorptive properties under different conditions, with a focus on the impact of pH, sorption kinetics, and equilibrium modeling [35,36,37,38,39,40,41,42,43,44]. Characterization of the material was carried out using FTIR and thermal analysis, which identified coordination between metal ions and the polymer’s functional groups. Further investigation of the polymer morphology through PEM, BET and NDR will be presented in future works to provide a more thorough understanding of the system’s behavior.

## 2. Materials and Methods

### 2.1. Materials

#### 2.1.1. Materials and Preparation

Ammonium perrhenate (NH_4_ReO_4_, ≥99%, analytical grade, Sigma-Aldrich, Taufkirchen, Germany) and sodium molybdate dihydrate (Na_2_MoO_4_·2H_2_O, ≥99%, analytical grade, Sigma-Aldrich, Taufkirchen, Germany) were used to prepare model solutions. Stock solutions were prepared at initial concentrations of 110 mg/L for each metal ion using double-distilled water. A 4 M hydrochloric acid solution (analytical grade, Sigma-Aldrich) was employed as the eluent. Interpolymer systems were studied in the intergel format, with each polymer gel placed in a separate semi-permeable pouch to ensure physical separation and contact-driven interaction of functional phases. Throughout the manuscript, the term “intergel” has been replaced with “interpolymer,” which more accurately describes the system composed of the poly(methacrylic acid) (PMAA) and poly(4-vinylpyridine) (P4VP) copolymer.

#### 2.1.2. Synthesis of Polymers and Preparation of Interpolymer Systems

Poly(methacrylic acid) (PMAA) polymer gel was synthesized as a lightly crosslinked polymer network using N,N′-methylenebisacrylamide (MBAA, ≥99%, Sigma-Aldrich, Taufkirchen, Germany) as the crosslinker and a K_2_S_2_O_8_–Na_2_S_2_O_3_ redox system (analytical grade, Sigma-Aldrich) as the initiator. To a 200 mL round-bottom flask were added: 5.00 g of methacrylic acid (MAA), 0.050 g of N,N′-methylenebisacrylamide, 1 mass% relative to MAA, 0.050 g of K_2_S_2_O_8_, and 0.050 g of Na_2_S_2_O_3_, each 1 mass% relative to MAA. 100 mL of deionized water was added, and the mixture was stirred until fully dissolved. The solution was degassed with nitrogen for 15 min. Polymerization was carried out at 25 °C for 24 h without stirring. The resulting gel was washed with water until it reached a neutral pH and then dried at 40 °C. The resulting polymer exhibited a swelling coefficient of Kₙ(PMAA) = 20.65 g/g.

Poly(4-vinylpyridine) (P4VP) polymer gel was prepared by crosslinking linear poly(4-vinylpyridine) (P4VP, Mw ~ 60,000, ≥99%, Sigma-Aldrich, Taufkirchen, Germany) with epichlorohydrin (≥99%, analytical grade, Sigma-Aldrich) in N,N-dimethylformamide (DMF, ≥99.8%, anhydrous, Sigma-Aldrich). Linear P4VP (1.00 g) was dissolved in anhydrous DMF (10–15 mL) at 25 °C under stirring until a clear solution was obtained (30–60 min). Epichlorohydrin was added at ECH:N_pyr_ = 0.30 (0.30 M ECH per 1.00 M pyridine nitrogen in P4VP), followed by NaOH at ~1.0 equiv per ECH (thus NaOH:N_pyr_ ≈ 0.30). Sodium hydroxide (NaOH) (NaOH, ≥97%, Emplura, Merck KGaA, Darmstadt, Germany) was introduced as a base to promote S_N_2 N-alkylation at the –CH_2_Cl group of ECH and to catalyze subsequent epoxide ring opening; it also neutralizes the HCl formed. NaOH was added as a DMF/H_2_O solution with ≤3–5 wt% water. The polymer and epichlorohydrin were stirred at 60 °C for 12 h (nitrogen blanket optional) until gelation was observed. Under these conditions, crosslinking proceeds via quaternization of the pyridine nitrogen by epichlorohydrin followed by interchain epoxide ring opening, yielding β-hydroxypropyl bridges (N^+^-CH_2_-CH(OH)-CH_2_-N^+^) between P4VP chains. The reaction was monitored by the change in the physical state of the mixture. The gel was first washed thoroughly with DMF and then with deionized water to remove unreacted species and soluble fractions (water replaced 2–3 times per day until the filtrate conductivity was <5–10 µS cm^−1^). The chloride counter-ion was exchanged to nitrate (3×, 1.0 M NaNO_3_, solid-to-liquid ≥ 1:10 *w*/*v*, 2 h per exchange at 25 °C), followed by rinsing to neutrality. Samples were dried under vacuum at 40–50 °C to constant mass. When required, the xerogel was gently ground and sieved to 130–150 µm. The swelling coefficient of the resulting hydrogel was Qₙ(P4VP) = 3.27 g/g. ECH is a strong alkylating (corrosive) agent, and DMF is toxic; all operations were carried out in a fume hood with appropriate PPE. ECH/DMF waste was collected separately and disposed of in accordance with institutional regulations.

Both polymers were obtained as transparent hydrogel films, which were subsequently dried and ground to irregular particles (0.3–0.5 mm) before use. The materials were soft and elastic and exhibited reversible swelling in water, consistent with typical hydrogel behavior. The dried particles displayed a porous, heterogeneous surface, facilitating efficient diffusion of aqueous ions into the polymer network. All reagents used were of analytical grade and were employed without additional purification.

### 2.2. Methods and Equipment

#### 2.2.1. Equipment and Analytical Methods

##### ICP-AES Analysis

Quantitative determination of rhenium and molybdenum was performed using an ICP-AES spectrometer (Shimadzu ICPE-9000, Kyoto, Japan). Calibration was carried out with certified standard solutions. The detection limits were 0.01 mg/L for Re(VII) and 0.05 mg/L for Mo(VI). For simplicity, throughout the manuscript, Re(VII) and Mo(VI) are used for rhenium and molybdenum ions, respectively. Their ionic forms, ReO_4_^−^ and MoO_4_^2−^, are consistently written with superscripts.

##### FTIR Spectroscopy

Infrared spectra were recorded on a JASCO FTIR spectrometer (Tokyo, Japan) in the range 4000–350 cm^−1^. Samples were prepared as KBr pellets (sample: KBr = 1:100). The spectra were analyzed to identify functional groups and evaluate complex formation.

##### Thermal Analysis (TGA/DSC)

Thermal properties were studied using a Netzsch TG 209 F3 Tarsus instrument (Selb, Germany). Measurements were carried out in a nitrogen atmosphere (50 mL/min) at a heating rate of 10 °C/min from 25 to 600 °C. Simultaneous TGA and DSC curves were obtained to evaluate mass loss, thermal stability, and energetic effects.

##### pH Control

During sorption and desorption experiments, pH was monitored using a pH200EM REX pH meter (Inesa Instrument Rex, Shanghai, China).

#### 2.2.2. Sorption Experiments

Throughout the manuscript, we have standardized the notation for rhenium and molybdenum ions. Re(VII) and Mo(VI) are now consistently used, and the ionic forms ReO_4_^−^ and MoO_4_^2−^ are written with superscripts, ensuring consistency throughout the manuscript. Batch sorption experiments were performed by mixing 0.10 g of dry PMAA–P4VP interpolymer with 50 mL of ReO_4_^−^ or MoO_4_^2−^ solutions in conical flasks, maintaining a temperature of 25 ± 1 °C. Stirring was done at 200 rpm until equilibrium was achieved. Throughout the experiments, the pH, temperature, and ionic strength were kept constant to ensure consistent conditions. The molar ratios of PMAA:P4VP ranged from 6:0 to 0:6.

Kinetic experiments were conducted with contact times ranging from 0.5 to 48 h. Samples of 5 mL were collected at pre-set intervals (0.5, 2.5, 6, 24, and 48 h) for analysis.

Isotherm experiments were carried out by varying the initial concentrations of rhenium and molybdenum between 10 and 300 mg/L, using a fixed sorbent mass of 0.10 g and a solution volume of 1000 mL. The systems were allowed to equilibrate for 48 h, after which the residual concentrations were measured using ICP-AES.

Sorption efficiency (η, %) was calculated as the percentage of metal ions removed from the solution, using the formula provided in Equation (1):

(1) $$\eta =\frac{C_{0}-C_{e}}{C_{0}}\times 100\%$$

C_0_ is the initial concentration and C_e_ is the equilibrium concentration (mg/L).

The equilibrium sorption capacity (q_e_, mg/g) was calculated using the equation shown below:

(2) $$q_{e}=\frac{\left(C_{0}-C_{e}\right)\times V}{m}$$

where C_0_ and C_e_ (mg/L) represent the initial and equilibrium concentrations of the metal ions, V (L) is the solution volume, and m (g) is the dry mass of the sorbent (0.10 g). This parameter allows for direct comparison with literature data.

To evaluate selectivity, the distribution coefficient (Kd, L·g^−1^) for each ion was calculated, along with the separation factor (β) for the Re/Mo pair under identical conditions:

(3) $$K_{d}=\frac{C_{0}-C_{e}}{C_{e}}\times \frac{V}{m}$$

where C_0_ and C_e_ (mg/L) are the initial and equilibrium concentrations, V (L) is the solution volume, and m (g) is the dry sorbent mass. Selectivity was assessed at C_0_ = 110 mg/L, V = 0.05 L, m = 0.10 g, and t = 48 h, unless stated otherwise. Results are presented as mean ± SD (*n* = 3).

To compare the selectivity of the system for different ions, the separation factor (selectivity coefficient) was determined as

β_Re/Mo_ = (K_d_,_Re_)/(K_d_,_Mo_) (4)

#### 2.2.3. Methodology of Desorption Experiments

Desorption was carried out using 4 M HCl. The desorption degree (R, %) was calculated using the following formula:

(5) $$R=\frac{m_{desorbed}}{m_{sorbed}}\times 100\%$$

#### 2.2.4. Binding Degree and Exchange Capacity

The binding degree (θ, %) was calculated as the ratio of the amount of metal ions bound at time t (qₜ) to the maximum sorption capacity (q_max_) obtained from the isotherm data:

(6) $$\theta =\frac{q_{t}}{q_{max}}\times 100\%$$

where $q_{t}$ was determined from experimental concentration differences between the initial and equilibrium solutions (C_0_ − C_t_) (mg/g), while $q_{\max}$ was obtained from Langmuir model fitting.

This parameter reflects the fraction of active sites occupied by metal oxyanions under given conditions, representing the proportion of coordination and electrostatic binding centers within the polymer framework of PMAA–P4VP that participate in ReO_4_^−^ and MoO_4_^2−^ complexation. Each value represents the mean of three independent measurements, and the relative standard deviation did not exceed 3%.

The effective dynamic exchange capacity (Q, mmol/g) was determined as

(7) $$Q=\frac{\nu_{sorb}}{m_{sorbent}}\times 100\%$$

#### 2.2.5. Swelling Properties

Swelling coefficients (Kₙ) were obtained gravimetrically:

(8) $$K_{n}=\frac{m_{2}-m_{1}}{m_{1}}$$

where m_1_ is the dry mass and m_2_ the swollen mass of the sample.

The high swelling coefficients (K_n_ = 20.65 g/g for PMAA and 3.27 g/g for P4VP) indicate a flexible, water-permeable network typical of hydrogel-type sorbents. This morphology facilitates ion diffusion and complex formation within the polymer. Additional structural characterization of the dried samples is being performed by X-ray diffraction (XRD) to evaluate crystallinity.

#### 2.2.6. Influence of pH on the Sorption Efficiency

The influence of pH was examined over the range 2.0–8.0 with a step of 1 unit, adjusted using 4 M HCl or NaOH. Other parameters (temperature, ionic strength, sorbent dosage, and contact time) were kept constant. Based on preliminary results, the ratios PAA:P4VP = 2:4 and PMAA:P4VP = 1:5 were selected as optimal. After 48 h of contact, residual concentrations of Re and Mo were determined. Data represent mean ± standard deviation from three replicates, with errors indicated on the graphs.

#### 2.2.7. Kinetics and Isotherm Modeling

The kinetics of sorption were evaluated using pseudo-first-order (PFO), pseudo-second-order (PSO), and intraparticle diffusion (Weber–Morris) models. These approaches allow the distinction between surface-controlled and diffusion-controlled processes.

##### Pseudo-First-Order (PFO) Model

The nonlinear form of the PFO model is expressed as

(9) $$q_{t}=q_{e}(1-e^{{-k}_{1}t})$$

where q_t_ is the amount of sorbed metal at time *t* (mg/g), *q_e_* is the equilibrium sorption capacity (mg/g), and *k*_1_, is the rate constant of the pseudo-first-order model (h^−1^).

This model is suitable for the sorption process controlled by surface interactions and under the condition of adsorption of metal ions on the polymer surface.

##### Pseudo-Second-Order (PSO) Model

The nonlinear PSO model is given by

(10) $$q_{t}=\frac{k_{2}q_{e}^{2}t}{1+k_{2}q_{e}t}$$

where k_2_ is the rate constant of the pseudo-second-order model (g mg^−1^ h^−1^)

The model is applicable to chemisorption and also to chemical reactions (binding of metal ions to polymer functional groups) that control sorption.

##### Intraparticle Diffusion (Weber–Morris) Model

The intraparticle diffusion model (Weber–Morris) is expressed as

(11) qt=kidt12+C

where $k_{id}$ is the intraparticle diffusion rate constant (mg·g−1h−12) and C is the intercept related to the boundary layer effect.

The model is applicable when the sorption rate is determined by diffusion within the polymer matrix, when the boundary layer resistance is significant, or when the sorption rate is limited by ion transport through the polymer network.

##### Model Selection and Interpretation

The pseudo-second-order (PSO) model demonstrated a good fit for most systems (R^2^ > 0.96). It showed that the chemisorption stage controls the process rate. For some compositions, the intraparticle diffusion (Weber–Morris) model provided additional information on the contribution of diffusion to the sorption process. The choice of kinetic model depends on the mechanism controlling sorption. The PFO model is applicable to surface-controlled adsorption, the PSO model is preferred when chemisorption predominates, and the Weber–Morris model is necessary for identifying diffusion-controlled processes in the polymer network.

##### Kinetic and Diffusion Modeling with Python and Google Colab

To analyze kinetics and diffusion within the Weber–Morris model, Python 3.12.5. code was written to process the experimental data. Google Colab 3.9., an environment for working with large datasets and visualizing results, was used to perform calculations and plot graphs. Pandas was used for data processing, Matplotlib 3.8.3. for plotting, and NumPy 1.26.4. for numerical calculations. The code was developed to calculate diffusion coefficients and assess the goodness of fit of the data to the Weber–Morris model. The resulting graphs allowed for a detailed analysis of the behavior of various interpolymer systems during the sorption of rhenium and molybdenum ions, to interpret changes in their sorption properties, and to identify trends depending on the system composition.

##### Sorption Isotherms

Sorption isotherms were obtained by varying the initial concentration of NH_4_ReO_4_ and Na_2_MoO_4_ between 10 and 250 mg L^−1^ for rhenium and for molybdenum, respectively, at 25 ± 1 °C. The solid-to-liquid ratio was 0.10 g per 50 mL of solution. All solutions were prepared using distilled water without any additional electrolyte adjustments, ensuring consistent ionic conditions throughout the experiments.

Equilibrium isotherm data analyzed using the Langmuir, Freundlich, and Dubinin–Radushkevich models. The Langmuir model assumes monolayer adsorption on a homogeneous surface:

(12) $$\frac{C_{e}}{q_{e}}=\frac{1}{q_{max}b}+\frac{C_{e}}{q_{max}}$$

where C_e_ is the equilibrium concentration (mg/L), q_e_ is the uptake (mg/g), q_max_ is the maximum capacity (mg/g), and b is the Langmuir constant (L/mg).

The Freundlich model, used for heterogeneous surfaces, is expressed as

(13) $${logq}_{e}={logK}_{f}+\frac{1}{n}{logC}_{e}$$

where K_f_ indicates the adsorption capacity and $\frac{1}{n}$ the heterogeneity factor.

Preliminary kinetic experiments were conducted to determine the equilibrium time for the sorption of Re(VII) and Mo(VI) ions on the PMAA–P4VP system at 298 K. The concentration of metal ions was monitored as a function of contact time in the range of 0.5–48 h. The sorption capacity increased rapidly during the first few hours (2–6 h) and then gradually approached a constant value. Equilibrium was achieved after approximately 48 h, beyond which no significant changes in ion uptake were observed. This equilibration time of 48 h was therefore used for all isotherm and thermodynamic measurements.

The sorption capacity at equilibrium, q_e_ (mg·g^−1^), was calculated from the concentration difference between the initial and equilibrium solutions using:

(14) $$q_{e}=\frac{\left(C_{0}-C_{e}\right)V}{m}$$

where C_0_ and C_e_ are the initial and equilibrium concentrations (mg·L^−1^), V is the solution volume (L), and m is the mass of the sorbent (g).

The maximum adsorption capacity (q_max_) was derived from nonlinear fitting of the Langmuir model, while b represents the equilibrium constant related to sorption energy.

Each data point corresponds to the mean of three parallel experiments, and the relative standard deviation did not exceed 3%. To ensure that equilibrium conditions were achieved, preliminary kinetic tests were performed over 0.5–48 h. The uptake values stabilized after 48 h, confirming that this time was sufficient to reach equilibrium and could be used consistently for isotherm evaluation.

Dubinin–Radushkevich (D–R) isotherm was fitted in its nonlinear form:

(15) $$q_{e}=q_{m}exp(-KDR\varepsilon^{2})$$

(16) ε=RTln(1+1Ce)

In the D–R analysis, C_e_ was expressed in mol L^−1^ of elemental Re (AAS basis), using M_Re_ = 186.207 g mol^−1^ and M_Mo_ = 95.96 g mol^−1^. The mean sorption energy was calculated as E = (2KDR)^−1/2^. Nonlinear fitting was used to avoid distortions of parameter estimates inherent to linearization. Calculations based on the corresponding salts: ammonium perrhenate (NH_4_ReO_4_, 268.2 g mol^−1^) and sodium molybdate (Na_2_MoO_4_·2H_2_O, 241.95 g mol^−1^)—yield the same trends, differing only by parameter scaling. Parameters were estimated by linearization in Microsoft Excel and by nonlinear regression in OriginPro 2023. Nonlinear fitting provided more accurate descriptions of sorption, yielding higher determination coefficients and lower deviations compared with linearized models.

All sorption experiments were performed in the absence of any background electrolyte to exclude competitive effects of foreign ions. The ionic strength of the solutions was therefore governed solely by the presence of ammonium perrhenate and sodium molybdate, corresponding to approximately 2.0 × 10^−3^–6.0 × 10^−3^ mol·L^−1^. This ensured identical ionic conditions for both systems and provided a consistent basis for comparison of rhenium and molybdenum sorption behavior.

#### 2.2.8. Mathematical Modeling and Validation of Adsorption Isotherms

##### Nonlinear Regression and Model Selection

To quantitatively describe the equilibrium sorption of Re(VII) and Mo(VI) oxyanions on the PMAA:P4VP interpolymer system, nonlinear forms of the Langmuir and, Freundlich isotherm models were applied. All calculations were carried out in OriginPro 2023 (OriginLab, Northampton, MA, USA) using the Nonlinear Curve Fit (NLFit) module, which allows fitting of user-defined and built-in functions. The optimization was performed using the Levenberg–Marquardt algorithm, a robust iterative method that combines the advantages of the Gauss–Newton and gradient-descent approaches to minimize the sum of squared errors (SSE) between the experimental (q_e_,_exp_) and calculated (q_e_,_calc_) equilibrium capacities:

(17) $$SSE=\sum_{i=1}^{n}{\left(q_{e,exp}^{\left(i\right)}\right.-\left.q_{e,calc}^{\left(i\right)}\right)}^{2}$$

Initial parameter estimates (q_max_, Kₗ, K_f_, and n) were generated automatically and refined manually until convergence criteria (|Δχ^2^| < 10^−6^) were satisfied. Parameter constraints were set to positive values, ensuring the physical meaning of adsorption constants.

The nonlinear forms of the applied models were expressed as

(18) $$Langmuir\;isotherm:q_{e}=\frac{q_{max}K_{L}C_{e}}{1+K_{L}C_{e}}$$

(19) Freundlich isotherm:qe=KfCe1n

##### Statistical Evaluation of Fit Quality

To assess the reliability and predictive accuracy of the nonlinear fits, several statistical indicators were evaluated:

Coefficient of determination (R^2^): measures the proportion of variance explained by the model.

Root Mean Square Error (RMSE): quantifies the mean deviation between experimental and calculated q_e_ values.

Reduced chi-square (χ^2^): evaluates the overall goodness-of-fit and parameter significance.

Akaike Information Criterion (AIC): used to compare models with different numbers of fitting parameters.

The best-fitting model was identified based on the highest R^2^ and lowest RMSE, χ^2^, and AIC values. Representative fitting parameters are summarized in Table 1.

##### Residual and Confidence Analysis

Residuals were plotted against fitted values to verify randomness and homoscedasticity. The absence of systematic trends confirmed the adequacy of the chosen nonlinear models. Confidence intervals at the 95% probability level were calculated for all parameters using covariance analysis of the data set. In addition, Q–Q plots and residual histograms generated in OriginPro confirmed the normal distribution of residuals, validating the statistical consistency of the model.

##### Model Comparison and Validation

Among the tested models, the Langmuir isotherm provided the best fit for both Re(VII) and Mo(VI) ions, indicating monolayer adsorption on energetically non-uniform surfaces with finite active sites. The Freundlich model, while less accurate statistically, adequately described systems exhibiting slight heterogeneity or multilayer sorption behavior. The results demonstrate that the PMAA:P4VP system follows a primarily Langmuir-type mechanism under the studied conditions.

#### 2.2.9. Determination of Distribution and Selectivity Coefficients

The distribution coefficients (K_d_, mL·g^−1^) for Re(VII) and Mo(VI) ions were calculated from the equilibrium concentrations using the following equation:

(20) $$K_{d}=\frac{\left(C_{0}-C_{e}\right)V}{C_{e}\times m}$$

where C_0_ and C_e_ (mg·L^−1^) are the initial and equilibrium concentrations of the metal ions, V (mL) is the volume of the solution, and m (g) is the mass of the dry sorbent.

The selectivity coefficient (β_Re/Mo_) was calculated as

(21) βReMo=KdReKdMo

These parameters were used to evaluate the preferential uptake of Re(VII) and Mo(VI) oxyanions by the PMAA–P4VP interpolymer system.

#### 2.2.10. Statistical Analysis

Each experiment was performed in triplicate, and the results are reported as mean ± standard deviation (SD). The standard deviation was calculated for each experimental condition to assess the variability of the data. Errors did not exceed 2%, ensuring the reliability of the measurements. Analysis of Variance (ANOVA) was used to assess statistical significance between experimental conditions, with a significance level of *p* ≤ 0.05. The statistical analysis of the experimental data was performed using Microsoft Excel 16. The data were analyzed by performing Analysis of Variance (ANOVA) to assess the significance of differences between the experimental conditions. Excel’s built-in functions were used to calculate the mean and standard deviation (SD) for each experimental condition. The results are expressed as mean ± SD, and error bars representing the standard deviation were applied to the figures. Statistical significance was considered at a significance level of *p* ≤ 0.05. ANOVA was performed using the Data Analysis Toolpak in Excel (Microsoft Office 16), and post hoc tests were used to compare individual means when appropriate.

#### 2.2.11. Morphology

The surface morphology of the polymer sorbents was investigated using scanning electron microscopy (SEM) JEOL JSM-6510 LA microscope (JEOL Ltd., Akishima, Tokyo, Japan) was employed, equipped with a tungsten filament electron gun using thermionic emission. Images were captured under low-vacuum conditions (<9 × 10^−3^ Pa) to reduce charging and minimize gas emission from the polymer samples. Secondary electrons were detected using the in-chamber secondary electron detector. SEM imaging was performed over a wide range of magnifications (3× to 300,000×), with a field of view of 7.7 mm at a working distance of 5 mm and 80 mm at a working distance of 30 mm. The accelerating voltage ranged from 0.5 kV to 30 kV, providing high-quality surface contrast, with an energy resolution of <140 eV (Mn K_β_) and a lower and an upper detector, enabling high-sensitivity detection of elements from B (Z = 5) to Cf (Z = 98). Samples were mounted on double-sided conductive tape and coated with a thin conductive layer under vacuum. The obtained SEM images of PMAK and P4VP before and after rhenium sorption allowed us to observe the surface porosity and changes caused by metal binding. The SEM analysis confirmed that both PMAA and P4VP hydrogels possess porous, heterogeneous surfaces, characteristic of lightly crosslinked polymer gels, which facilitate efficient diffusion of metal ions into the network and enable the formation of coordination complexes.

## 3. Results and Discussion

### 3.1. Effect of Interpolymer Composition on Rhenium Sorption

**Figure 1:**
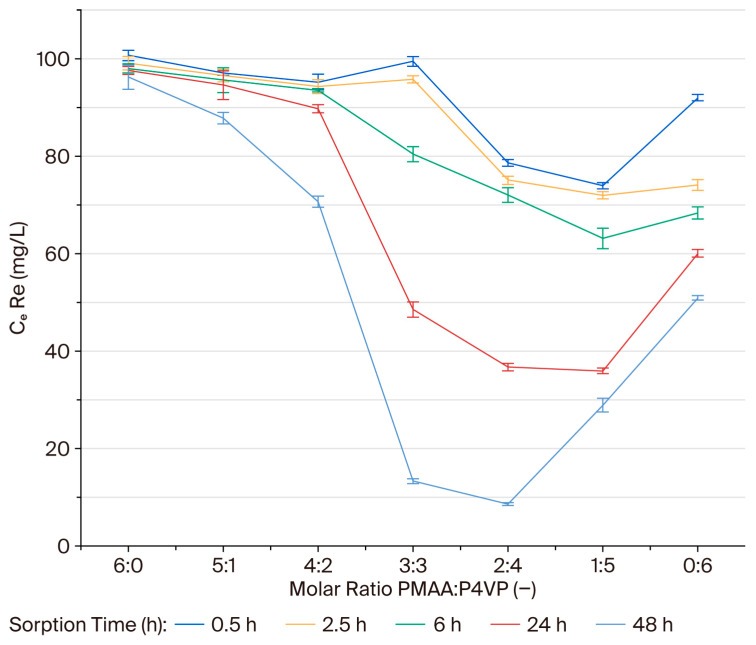
Residual concentration of rhenium ions (C_e_, mg/L) as a function of the PMAA:P4VP molar ratio at different contact times (0.5–48 h). Data points represent the mean values from three independent experiments, with error bars showing the standard deviations (mean ± SD, *n* = 3), where SD ≤ 2%.

The influence of interpolymer composition on the sorption of rhenium ions was investigated using PMAA:P4VP systems with molar ratios ranging from 6:0 to 0:6. The equilibrium residual concentrations of ReO_4_^−^ ions were measured after 48 h of contact. The results indicate that rhenium sorption efficiency is significantly affected by the polymer composition. Systems with molar ratios of 2:4 and 3:3 exhibited the lowest residual concentrations (C_e_ = 8.6 and 13.3 mg/L, respectively), demonstrating a higher affinity for ReO_4_^−^ anions. In contrast, systems dominated by a single functional component—either acidic (6:0, 5:1) or basic (1:5, 0:6)—showed notably higher equilibrium concentrations, indicating reduced interaction capacity. To illustrate these trends, residual concentrations were plotted as a function of polymer composition (Figure 1). The 2:4 and 3:3 systems displayed the highest affinity for ReO_4_^−^, confirming that optimal sorption occurs when both carboxyl (–COOH) and pyridyl nitrogen groups (–C_5_H_4_N) are present in similar proportions. This balance ensures simultaneous electrostatic attraction and donor–acceptor coordination with rhenium oxyanions. The cooperative effect between the carboxyl and pyridine functionalities enhances the accessibility and reactivity of sorption centers, leading to improved uptake efficiency. Therefore, compositions with balanced or pyridine-enriched structures (particularly 2:4 and 3:3) can be considered the most effective for selective and stable recovery of rhenium from aqueous media.

### 3.2. Effect of the Interpolymer Composition on Molybdenum Sorption

**Figure 2:**
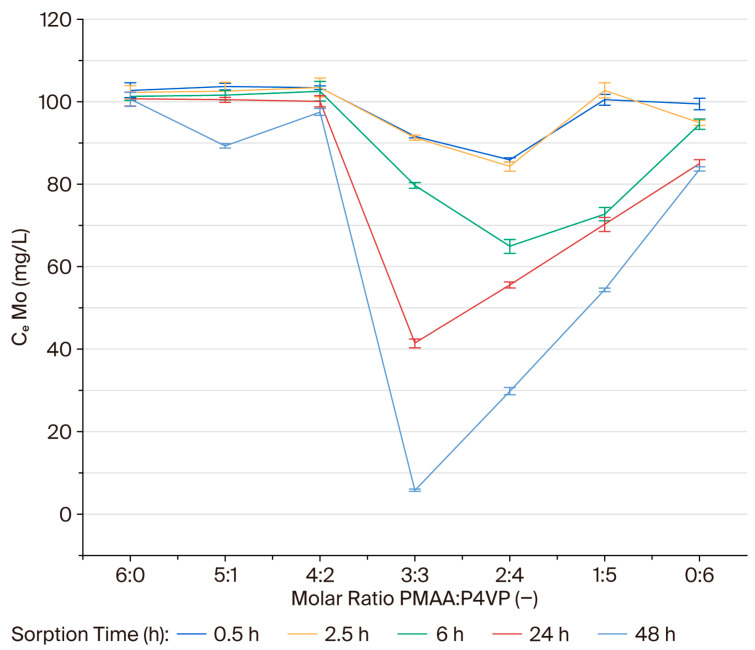
Residual concentration of molybdenum ions (Ce, mg/L) as a function of the PMAA:P4VP molar ratio at different contact times (0.5–48 h). Each data point represents the mean of three replicate measurements, with error bars showing standard deviations (mean ± SD, *n* = 3), where SD ≤ 2%.

Sorption of ${MoO}_{4}^{2-}$ ions was investigated under conditions identical to those used for rhenium: initial concentration 110 mg/L, controlled pH and ionic strength, and contact times up to 48 h. Residual concentrations were determined at regular intervals. Sorption of molybdate ions by the PMAA–P4VP interpolymer systems was examined under quasi-equilibrium conditions at predefined contact times. Although the data were recorded at several time points, they primarily reflect the approach to equilibrium rather than kinetic behavior. At the early stages (0.5–2.5 h), most systems exhibited limited uptake, with residual concentrations (C_e_) exceeding 100 mg/L. The 3:3 and 2:4 compositions, however, already showed a noticeable decrease (91.60 and 85.90 mg/L, respectively). After 48 h, the 3:3 composition achieved the lowest residual concentration (C_e_ = 5.80 mg/L), indicating the highest overall sorption efficiency, whereas the 2:4 system reached C_e_ = 29.80 mg/L. In contrast, compositions dominated by a single polymer component (6:0, 0:6, 5:1, 4:2, 1:5) maintained higher C_e_ values (54–100 mg/L). These results indicate that balanced interpolymer compositions, with comparable proportions of PMAA and P4VP, provide the most effective binding of MoO_4_^2−^ ions under equilibrium conditions. To visualize these trends, residual concentrations were plotted as a function of polymer composition (Figure 2). The plots clearly show limited uptake during the initial contact period (0.5–2.5 h), followed by a notable divergence in sorption efficiency at longer times. As equilibrium is approached, the 2:4 and 3:3 systems exhibit the steepest decline in residual molybdenum concentration, confirming their superior affinity for MoO_4_^2−^ ions. These results suggest that the balanced acid–base functionality within the interpolymer network, combined with adequate contact time, promotes complex formation and enhances selectivity. Hence, the 2:4 and 3:3 systems can be considered the most effective compositions for practical molybdenum recovery from aqueous media.

Further analysis of the kinetic behavior reveals that the sorption process is governed by both surface interaction and intraparticle diffusion mechanisms. At early contact times (0.5–2.5 h), the P4VP-rich systems show fast uptake of MoO_4_^2−^ ions, which can be attributed to the interaction of pyridyl nitrogen groups with the oxyanion. As equilibrium is approached, the 3:3 and 2:4 systems, with balanced functional groups, show the highest sorption efficiency, which can be explained by a slower diffusion process that allows better access of the ions to deeper coordination sites within the polymer network.

### 3.3. Mechanistic Interpretation of Sorption Kinetics

Based on the results of the kinetic studies, the sorption process can be described as follows: it begins with surface adsorption, followed by intraparticle diffusion (IPD). Active ion uptake occurs within the time interval of 0.5–2.5 h for the interpolymer systems enriched in P4VP. In particular, the uptake of the ReO_4_^−^ anion is most pronounced for the PMAA:P4VP = 1:5 system, while the absorption of the MoO_4_^2−^ anion is characteristic of the PMAA:P4VP = 2:4 composition. This effect is largely attributed to the nitrogen atoms in the P4VP polymer, which can form coordination bonds through their lone pairs of electrons. In the 1:5 composition, where pyridyl segments dominate the binding sites for ReO_4_^−^, sorption initiates with high efficiency. Pyridyl groups act as electron-donating coordination centers, binding metal ions and facilitating their rapid uptake during the early stages of sorption. Over the next 24–48 h, the system gradually approaches equilibrium. As equilibrium is reached, the sorption curve flattens, reflecting the transition of the system into a stable state. In contrast, mixtures combining both carboxyl and pyridyl functionalities—particularly the compositions with PMAA:P4VP ratios of 3:3 and 2:4—exhibit a more uniform sorption rate and, as a result, achieve the highest overall efficiency. This is likely due to the optimal balance between the carboxyl and pyridyl groups, whose cooperative interaction promotes faster metal-ion transport and enhances coordination within the polymer matrix. They exhibit rapid initial absorption but low final efficiency due to a less optimal distribution of functional groups. The kinetics of sorption in these systems depend on the surface interactions and the depth of coordination sites available in the polymer matrix. Although the 1:5 system for the Re-oxyanion and the 2:4 system for the Mo-oxyanion exhibit high initial sorption rates, balanced systems (2:4 and 3:3) achieve the highest efficiency due to slower, more efficient diffusion and coordination processes.

### 3.4. Sorption Efficiency (η) of Rhenium Ions on the PMAA–P4VP Interpolymer System

Sorption efficiency for ReO_4_^−^ (η, %) was calculated for all compositions over 48 h. At early times (0.5–6 h), measurable uptake is observed only in P4VP-rich systems. At 0.5 h, the highest η values occur for 1:5, 2:4, and 0:6 (28.38%, 23.85%, and 10.89%, respectively). After 6 h, 1:5 and 0:6 remain leading (η > 30%), while 2:4 and 3:3 continue to increase steadily. After 24 h, the sorption efficiency (η) increased for all compositions, with the highest values observed for 1:5 (65.19%) and 2:4 (39.18%). After 48 h, near-complete removal of rhenium was achieved by the 2:4 and 3:3 systems (η = 91.67% and 87.12%, respectively). The 1:5 and 0:6 systems also remained effective (η = 71.99% and 50.67%), demonstrating the role of pyridyl sites in ReO_4_^−^ coordination. Overall, balanced or P4VP-rich matrices provide the most efficient rhenium extraction under the studied conditions. Rhenium sorption behavior showed similar trends to molybdenum. At early contact times (0.5–2.5 h), the P4VP-enriched systems (1:5 and 0:6) exhibited higher sorption efficiencies for ReO_4_^−^ ions. However, as equilibrium approached (24–48 h), these systems demonstrated a plateau in removal efficiency, and the 2:4 and 3:3 systems, with a balanced composition of both functional groups, achieved the highest removal efficiencies. The final sorption capacities at equilibrium for the 2:4 and 3:3 systems were 91.67% and 87.12%, respectively. These results suggest that while P4VP-rich systems show initial high uptake, the overall efficiency at equilibrium is greater in systems with balanced acid–base functionality.

### 3.5. Sorption Efficiency of the PMAA–P4VP Interpolymer System for Molybdenum Ions

**Figure 3:**
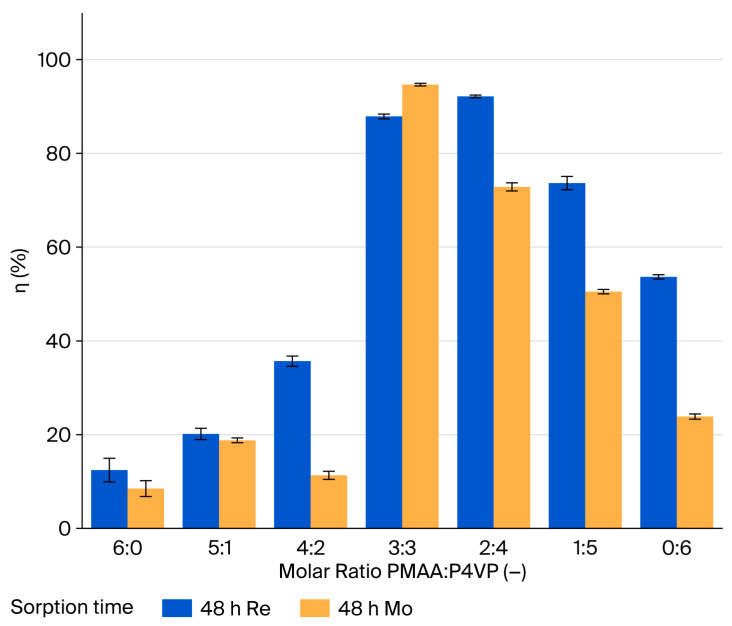
Sorption efficiency (η, %) of rhenium and molybdenum ions by PMAA–P4VP interpolymer systems at different molar ratios (sorption time = 48 h). η = ((C_0_ − C_e_)/C_0_) × 100%, where C_0_ and C_e_ are the initial and equilibrium concentrations (mg/L). Experimental conditions: C_0_(Re(VII)) = C_0_(Mo(VI)) = 110 mg/L; solution volume = 50 mL; sorbent dose = 0.10 g; temperature = 25 ± 1 °C; pH maintained at 5.2. Each point represents the mean of three independent measurements; error bars indicate standard deviation (mean ± SD (*n* = 3), SD ≤ 2%).

Sorption efficiency for ${MoO}_{4}^{2-}$ (η, %) was determined for all compositions over 48 h. This represents the percentage of metal ions removed from the solution, confirming that η corresponds to the removal efficiency (Removal-%). During the initial 0.5–2.5 h, the uptake is generally low; however, the 3:3 (η = 20.02–21.51%) and 2:4 (η = 14.71–14.99%) systems exhibit significant reductions in residual molybdenum concentrations. After 6 h, the 2:4 system shows a sharp increase in sorption efficiency (η = 39.55%), while the 3:3 system reaches 25.80%. The 1:5 and 0:6 compositions display moderate uptake (η = 32.23% and 11.95%, respectively). To further explore the sorption mechanisms, a kinetic analysis was conducted to evaluate the uptake behavior of MoO_4_^2−^ ions as a function of time. At early contact times (0.5–2.5 h), the P4VP-enriched systems (1:5, 0:6) exhibited higher sorption efficiencies compared to other systems. However, over time, the sorption efficiency of these systems plateaued. In contrast, the 2:4 and 3:3 systems, which have a more balanced acid–base composition, demonstrated a slower but steady increase in sorption efficiency, ultimately achieving the highest removal efficiencies after 48 h. These results suggest that while P4VP-rich systems show initial high uptake, the systems with balanced functional groups (2:4 and 3:3) offer higher overall efficiency at equilibrium. After 24 h, 3:3 shows a pronounced increase (η = 76.43%), followed by 2:4 (η = 49.80%), 1:5 (η = 34.60%), and 0:6 (η = 20.76%). Maximum efficiencies at 48 h are recorded for 3:3 (η = 94.60%) and 2:4 (η = 72.25%); other compositions remain moderate or low, without signs of synergistic enhancement. Research results and a comparative histogram for Re and Mo (Figure 3) confirm the superiority of 2:4 and 3:3, attributable to cooperative interactions between acidic and basic groups. P4VP-rich systems (1:5, 0:6) are intermediate, whereas PMAA-rich matrices (6:0, 5:1) are least effective.

### 3.6. Binding Degree (θ) of Rhenium Ions by the PMAA–P4VP Interpolymer System

**Figure 4:**
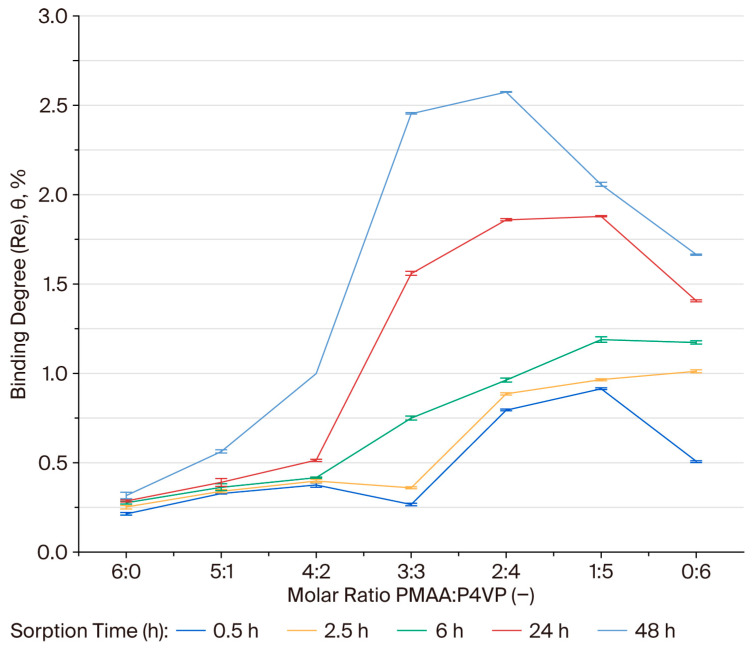
Binding degree (θ, %) of rhenium ions by PMAA–P4VP interpolymer systems as a function of molar ratio and contact time. Initial concentration of Re(VII): 110 mg/L; solution volume: 50 mL; sorbent dose: 0.10 g; temperature: 25 ± 1 °C; pH = 5.2. Each value represents the mean of three measurements; error bars show standard deviation (mean ± SD (*n* = 3), SD ≤ 2%).

The binding degree (θ, %) was calculated to evaluate the specific coordination of ReO_4_^−^ ions within the polymer network. The binding degree for rhenium ions was assessed for PMAA–P4VP systems with various molar ratios. During the first 0.5–2.5 h, the binding degree (θ) remained low in most systems, with early activation observed in the 2:4, 1:5, and 0:6 compositions, suggesting the involvement of pyridyl sites in the initial coordination. By 6 h, system 1:5 exhibited the highest θ (1.18%), followed by 0:6 (1.17%) and 2:4 (0.96%), while 3:3 began to increase (0.75%). After 24 h, the binding degree (θ) increased significantly for the 1:5 (1.87%) and 2:4 (1.85%) systems. After 48 h, the 2:4 and 3:3 systems reached the highest binding degrees (2.57% and 2.45%, respectively), indicating an optimal balance between carboxyl and pyridyl groups for rhenium coordination. The 1:5 (2.05%) and 0:6 (1.66%) systems also maintained considerable binding capacity, while PMAA-dominated compositions (6:0, 5:1, 4:2) showed lower binding, indicating that carboxyl groups alone are insufficient for strong ReO_4_^−^ complexation under these conditions. The plots in Figure 4 illustrate these equilibrium trends, highlighting the clear dominance of the 2:4 and 3:3 compositions.

### 3.7. Binding Degree (θ) of Molybdenum Ions by the PMAA–P4VP Interpolymer System

**Figure 5:**
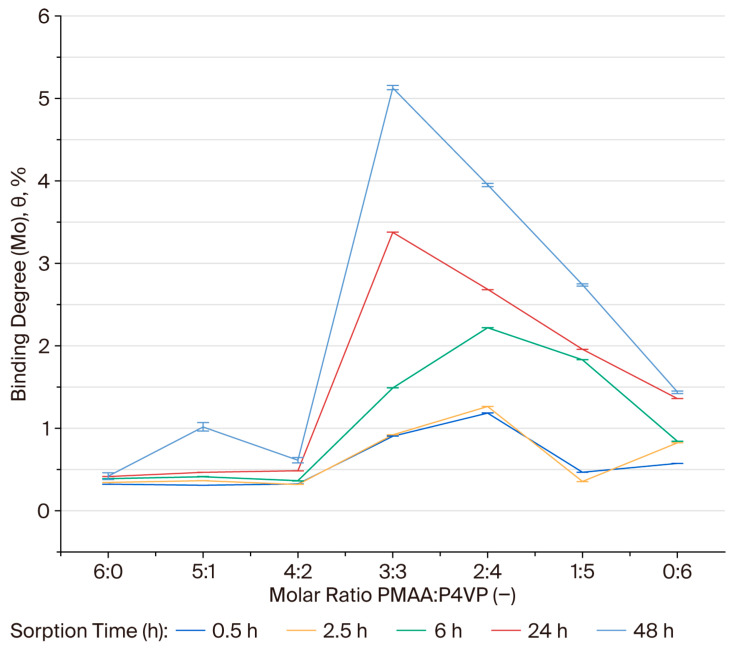
Binding degree (θ, %) of molybdenum ions as a function of PMAA:P4VP molar ratio and contact time. Experimental conditions: initial Mo(VI) concentration of 110 mg/L; solution volume of 50 mL; sorbent dose of 0.10 g; temperature of 25 ± 1 °C; pH = 5.2. Error bars represent standard deviation (mean ± SD (*n* = 3), SD ≤ 2%).

The binding degrees for Mo(VI) were calculated for each composition over a 48 h period. The binding degree (θ, %) for molybdenum follows a similar compositional trend to that observed for rhenium. The 3:3 and 2:4 systems exhibited the highest binding degrees (5.13% and 3.95% after 48 h), indicating effective coordination between MoO_4_^2−^ oxyanions and both carboxyl and pyridyl functional groups. The binding degree (θ, %) for molybdenum ions was evaluated for each composition over 48 h. The 3:3 and 2:4 compositions exhibited the highest binding degrees (5.13% and 3.95%, respectively), indicating effective coordination between MoO_4_^2−^ oxyanions and both carboxyl and pyridyl functional groups. These results parallel the trends observed for rhenium sorption, confirming the critical role of balanced acid-base functionalities in enhancing coordination. The equilibrium trends for Mo(VI) sorption are illustrated in Figure 5, with the 3:3 and 2:4 compositions showing the most pronounced sorption efficiency.

The gradual increase in the binding degree after 24 h suggests that the sorption process is not solely surface-controlled but also involves diffusion and structural rearrangement within the interpolymer network. Swelling of the interpolymer network increases the mobility of functional groups, improving the accessibility of internal –COOH and pyridyl sites, which leads to a higher proportion of coordination centers being occupied. Such delayed activation effects are typical of interpenetrating or interpolymer systems, where ion transport is initially restricted during the early stages of contact.

### 3.8. Effective Dynamic Exchange Capacity of the PMAA–P4VP Interpolymer System Toward Rhenium Ions

**Figure 6:**
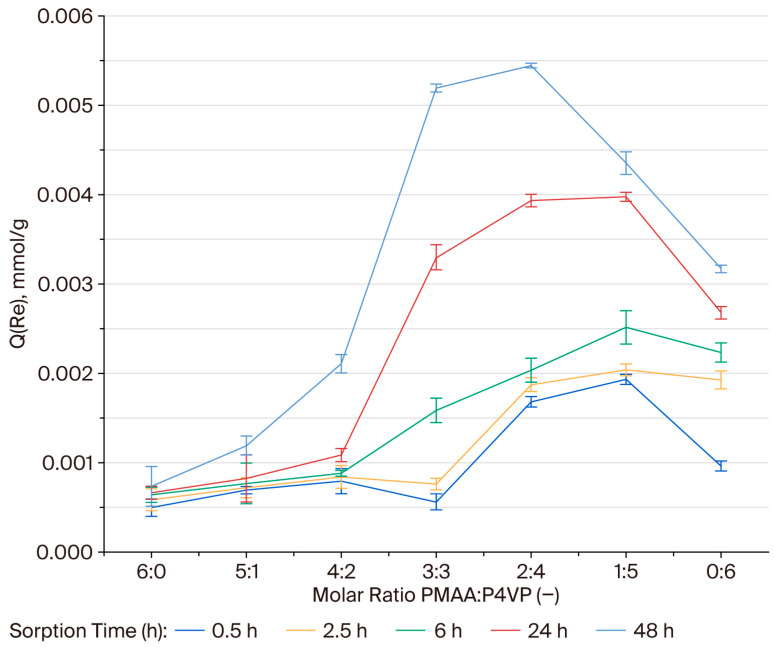
Effective dynamic exchange capacity (Q, mmol/g) of the PMAA–P4VP interpolymer system toward rhenium ions as a function of molar ratio and contact time. Conditions: Re(VII), 110 mg/L; solution volume: 50 mL; sorbent dose: 0.10 g; temperature: 25 ± 1 °C; pH = 5.2. Error bars represent the standard deviation (mean ± SD, *n* = 3), with SD ≤ 2%.

The effective dynamic exchange capacity (Q, mmol/g) was determined to assess the ion-exchange performance of the PMAA:P4VP systems in the sorption of ReO_4_^−^. During the initial stages (0.5–2.5 h), the Q values were generally low (<0.002 mmol/g), with the highest activity observed in the 2:4, 1:5, and 0:6 compositions, suggesting a rapid involvement of pyridyl sites. After 6 h, the Q values increased in the 1:5 (0.00204 mmol/g), 0:6 (0.00192 mmol/g), 3:3 (0.00076 mmol/g), and 2:4 (0.00187 mmol/g) systems. After 24 h, the ion-exchange process intensified, reaching 0.0039 mmol/g for 1:5, 0.003 mmol/g for 2:4, 0.0026 mmol/g for 0:6, and 0.00329 mmol/g for 3:3. After 48 h, the highest exchange capacities were observed for the 2:4 (0.0054 mmol/g) and 3:3 (0.0051 mmol/g) compositions. The 1:5 (0.00435 mmol/g) and 0:6 (0.003 mmol/g) systems also demonstrated considerable activity, while PMAA-rich compositions (6:0, 5:1, 4:2) remained less effective (Q < 0.003 mmol/g). Figure 6 illustrates the dependence of Q on polymer ratio and time. The 2:4 and 3:3 systems reached the most pronounced values at 48 h, confirming the cooperative contribution of pyridyl and carboxyl groups. The steepest increases in Q occurred between 24 and 48 h, indicating a delayed but sustained exchange process.

### 3.9. Effective Dynamic Exchange Capacity of the PMAA–P4VP Interpolymer System Toward Molybdenum Ions

**Figure 7:**
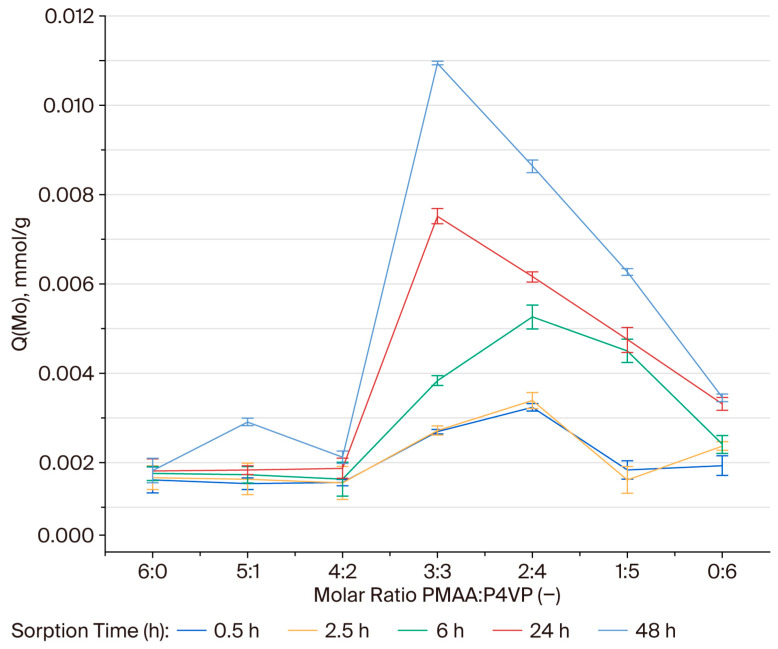
Effective dynamic exchange capacity (Q, mmol/g) of the PMAA–P4VP interpolymer system for molybdenum ions as a function of molar ratio and contact time. Experimental conditions: Mo(VI) concentration: 110 mg/L; solution volume: 50 mL; sorbent dose: 0.10 g; temperature: 25 ± 1 °C; pH = 5.2. Error bars represent standard deviation (mean ± SD (*n* = 3), SD ≤ 2%).

At 0.5–2.5 h, Q values remained below 0.003 mmol/g. By 6 h, 2:4 showed a sharp increase (0.0026 mmol/g), while 3:3 reached 0.0019. After 24 h, 3:3 achieved the highest value (0.00567 mmol/g), followed by 2:4 (0.0041 mmol/g). At 48 h, maxima were observed in 3:3 (0.01 mmol/g) and 2:4 (0.0080 mmol/g). The 1:5 (0.0057 mmol/g) and 0:6 (0.0027 mmol/g) systems showed moderate performance, while PMAA-rich matrices remained ineffective (Q < 0.0030 mmol/g). Figure 7 confirms these observations. The most significant increases occurred between 24 and 48 h, particularly in 3:3 and 2:4, reflecting the progressive nature of ${MoO}_{4}^{2-}$ complexation.

### 3.10. Effect of pH on Sorption Efficiency of Rhenium and Molybdenum Ions

The influence of pH on sorption efficiency was examined at pH values of 2, 4, 8, and 10, using an initial concentration of 110 mg/L for both Re(VII) and Mo(VI). Residual concentrations were measured after 0.5–48 h. Sorption was most effective under acidic conditions. At pH 2, the residual concentrations decreased to 58.34 mg/L for ReO_4_^−^ (≈61% removal) and 80.61 mg/L for MoO_4_^2−^ (≈46%), whereas at pH 4 the efficiency slightly declined, particularly for molybdenum. In neutral and alkaline media (pH 8–10), the residual concentrations remained above 97 mg/L for both metals, reflecting competition from hydroxide ions and the reduced electrostatic attraction between the polymer and oxyanions.

**Figure 8:**
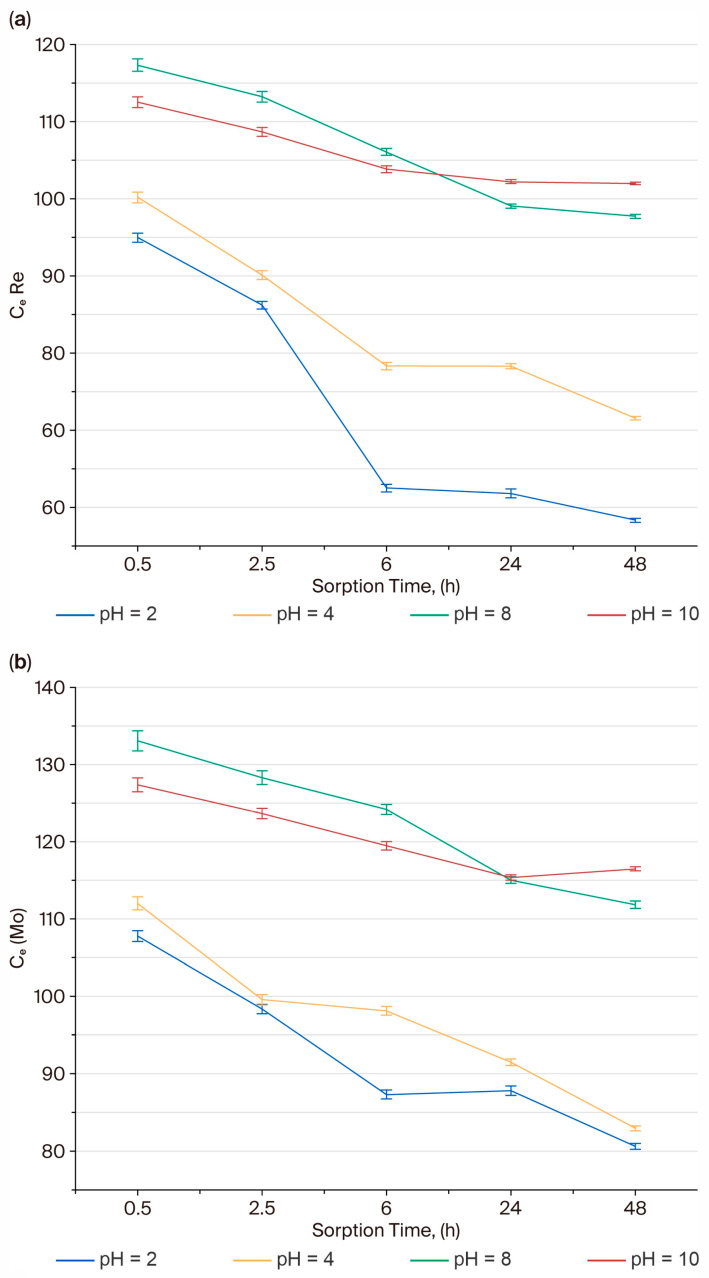
(**a**) Residual concentration of rhenium ions (${ReO}_{4}^{-}$, mg/L) in solution as a function of contact time at pH 2, 4, 8, and 10 using the PMAA–P4VP interpolymer system. (**b**) Residual concentration of molybdenum ions (MoO_4_^2−^, mg/L) under the same conditions. Initial concentrations: 110 mg/L each; solution volume: 50 mL; sorbent dose: 0.10 g; temperature: 25 ± 1 °C. Error bars represent the standard deviation (mean ± SD, *n* = 3), with SD ≤ 2%.

These trends reflect the protonation–deprotonation behavior of the polymer’s functional groups. Under acidic conditions, carboxyl groups (–COOH) are mostly protonated, reducing intramolecular repulsion between –COO^−^ sites, while pyridyl nitrogen atoms (–C_5_H_5_N^+^) become positively charged, promoting electrostatic attraction toward ReO_4_^−^ and MoO_4_^2−^. As pH increases, both groups undergo deprotonation, weakening the interaction and facilitating desorption. Figure 8a,b show the dependence of residual concentration (C_e_, mg/L) on pH. Minimum C_e_ values (i.e., maximum sorption) were observed at pH 2–4, confirming that acidic conditions favor coordination and electrostatic binding of Re(VII) and Mo(VI) oxyanions in the PMAA–P4VP interpolymer system.

As seen from the plots, where the decrease in residual concentration with increasing pH confirms that protonated pyridyl sites are responsible for metal uptake, the sorption efficiency markedly decreases with pH, indicating that acidic conditions (pH 2–4) favor the protonation of pyridyl and carboxyl groups and thereby enhance the uptake of rhenium and molybdenum ions. The dependence of sorption efficiency on pH can be explained by the speciation of Re(VII) and Mo(VI) ions and the protonation state of the polymer functional groups. Rhenium remains in solution mainly as ReO_4_^−^ throughout the studied range (pH 2–10); however, at higher pH values, the pyridine groups of P4VP become deprotonated, reducing electrostatic attraction toward ReO_4_^−^. In contrast, molybdenum exhibits pronounced pH-dependent speciation. At pH 2–5, poly-molybdate species (e.g., Mo_7_O_24_^6−^, HMoO_4_^−^) predominate, while at pH 6–8 the equilibrium shifts toward the monomeric MoO_4_^2−^ ion, which interacts most effectively with protonated sites of PMAA and P4VP. Above pH 8, competition from hydroxide ions and further deprotonation of carboxyl groups hinder anion binding, leading to the observed decrease in sorption efficiency. The equilibrium adsorption capacities (q_e_, mg g^−1^) were calculated using the equation q_e_ = (C_0_–C_e_) × V/m, where C_0_ and C_e_ are the initial and equilibrium concentrations (mg L^−1^), V is the solution volume (L), and m is the mass of the dry polymer (g).

**Figure 9:**
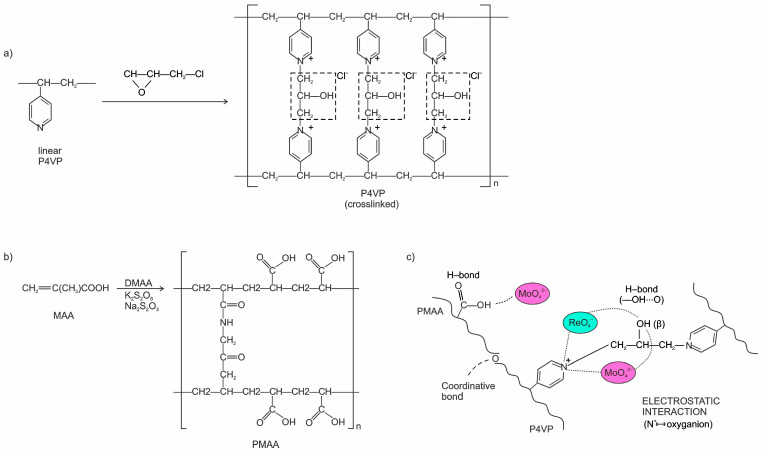
Schematic representation of (**a**) P4VP crosslinking with ECH, (**b**) PMAA formation, and (**c**) possible interaction mechanisms of PMAA–P4VP with MoO_4_^2−^ and ReO_4_^−^ ions via coordinative, hydrogen, and electrostatic interactions. Note: The ionic species (ReO_4_^−^ and MoO_4_^2−^) are shown in simplified schematic form for clarity; in reality, their coordination and interaction environments within the polymer matrix are more complex.

In DMF (N,N-dimethylformamide) and NaOH (sodium hydroxide), epichlorohydrin (ECH, C_3_H_5_ClO) acts as a bifunctional alkylating agent. In the first step, the nitrogen of the pyridine ring in P4VP substitutes chloride at the –CH_2_Cl fragment of ECH via an S_N_2 mechanism, forming an N-(2,3-epoxypropyl)pyridinium center (quaternary nitrogen, Cl^−^ as the counter-ion; the epoxide remains intact and is opened in the next step). In the second step, the epoxide ring of this substituent undergoes nucleophilic opening by the pyridine nitrogen of a neighboring macromolecular chain, yielding a β-hydroxypropyl bridge N^+^–CH_2_–CH(OH)–CH_2_–N^+^ between chains (Figure 9a). This pathway is consistent with the appearance of an alcohol group within the bridge, an increased fraction of quaternary N^+^ centers, and the cationic nature of the network.

The FTIR spectra of crosslinked P4VP agree with the proposed mechanism. In the purified gel, a broad O–H band near ~3400 cm^−1^ is observed, reflecting the β-hydroxy group in the bridge, together with a distinct C–O band near ~1060 cm^−1^ characteristic of the alcoholic linkage in the –CH_2_–CH(OH)–CH_2_– fragment. At the same time, the relative contribution of aliphatic C–H stretching at 2926 and 2855 cm^−1^ increases with respect to the ring region around ~1600 cm^−1^, and the profile in 1600–1550 cm^−1^ (ν(C=N)/pyridine skeletal vibrations) changes due to a higher fraction of pyridinium (N^+^) centers. Epoxide bands at ~910–830 cm^−1^ are not observed in the purified sample, which is consistent with whole oxirane ring opening during crosslinking.

Alternative crosslinking pathways through C-functionalization of the aromatic ring appear unlikely under DMF/NaOH conditions. Because they typically require strongly acidic media and/or Lewis acid catalysts. Reactions at the carbon of the main –CH_2_–CH– backbone (radical or electrophilic) are also not expected at the applied temperature and in this solvent/base system. The increase in β-hydroxypropyl N^+^ bridges is diagnosed by the growth of the C–O (~1060 cm^−1^) and O–H (~3400 cm^−1^) bands accompanied by a higher aliphatic contribution (2926/2855 cm^−1^), which is consistent with the formation of a denser cationic network.

**Figure 10:**
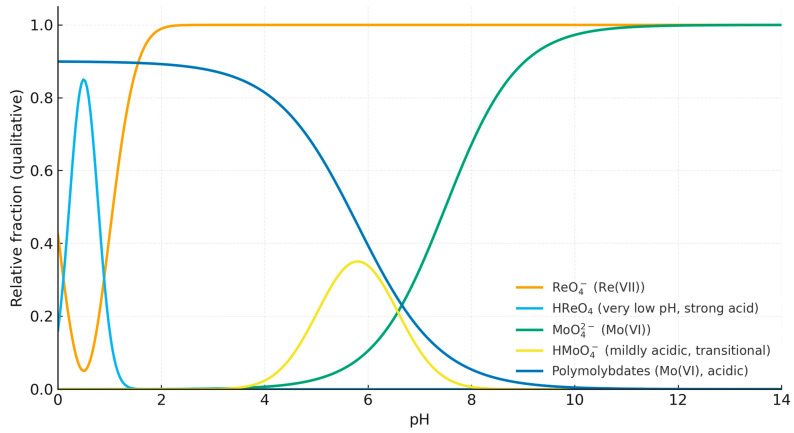
Speciation diagram of rhenium and molybdenum oxyanions in aqueous solution as a function of pH, indicating the dominant forms of Re(VII) and Mo(VI).

The observed variations in sorption behavior are consistent with the known aqueous speciation of Re(VII) and Mo(VI) oxyanions. Rhenium exists predominantly as the stable monomeric perrhenate ion (ReO_4_^−^) across a wide pH range (≈2–10), whereas molybdenum shows pH-dependent transformations: polymolybdate species (e.g., Mo_7_O_24_^6−^, HMo_7_O_24_^5−^) prevail under acidic conditions, HMoO_4_^−^ may appear in mildly acidic media, and monomeric MoO_4_^2−^ dominates near neutral to alkaline pH [45,46]. These equilibria affect the availability and charge of oxyanions and thus their association with the cationic P4VP–N^+^ sites (electrostatic, outer-sphere) and hydrogen-bond donors in PMAA (–COOH under acidic conditions) within the PMAA–P4VP network. The observed pH dependence of sorption is therefore consistent with both the protonation–deprotonation of functional groups in the polymer system and the aqueous speciation of Re(VII) and Mo(VI). The speciation diagram (Figure 10) indicates that ReO_4_^−^ and MoO_4_^2−^ are the dominant species in acidic-to-near-neutral and neutral-to-alkaline media, respectively; at higher pH, decreased sorption is rationalized by loss of hydrogen bonding from PMAA (–COOH → –COO^−^) and increased ionic-strength/competition effects, rather than by the formation of inner-sphere hydroxo complexes.

To highlight the competitiveness of the synthesized PMAA–P4VP system, the obtained equilibrium adsorption capacities were compared with data from recent literature on polymeric, hybrid, and inorganic sorbents used for the extraction of Re(VII) and Mo(VI) oxyanions. Table 2 summarizes the reported adsorption capacities (q_e_ or q_max_) along with operational pH and kinetic characteristics. The PMAA–P4VP copolymer exhibits balanced performance, achieving 48.6 mg g^−1^ for Re(VII) and 42.7 mg g^−1^ for Mo(VI) at near-neutral pH ≈ 5.2, which compares favorably with commercial anion-exchange resins (Purolite A170, 166.7 mg g^−1^) and inorganic adsorbents (NH_4_HCO_3_–Al_2_O_3_, 1.94 mg g^−1^) operating under much stronger acidic conditions. These results indicate that the PMAA–P4VP interpolymer system combines moderate adsorption capacity with good regenerability under environmentally mild conditions. As shown in Table 2, the sorption capacity of the PMAA–P4VP system is comparable to or exceeds that of many established polymeric and inorganic sorbents. Its effective performance under mildly acidic conditions (pH ≈ 5) and stability during regeneration cycles provide a practical advantage for the hydrometallurgical recovery of Re(VII) and Mo(VI).

As seen from Table 2, the sorption capacity of the PMAA–P4VP system is comparable to or higher than that of many known polymeric and inorganic materials. Its operation under mild acidic conditions (pH ≈ 5) and stability during regeneration cycles provide a practical advantage for hydrometallurgical recovery of Re(VII) and Mo(VI).

### 3.11. Selectivity of Re(VII) and Mo(VI) Sorption

The selectivity of the PMAA–P4VP interpolymer system toward Re(VII) and Mo(VI) oxyanions was assessed under optimal conditions (pH ≈ 5.2, C_0_ = 110 mg·L^−1^, 0.10 g polymer, 298 K). Both ions were present simultaneously in solution to evaluate their competitive uptake. As observed from the equilibrium data, rhenium was preferentially adsorbed, indicating a stronger interaction of its monovalent oxyanion (ReO_4_^−^) with the protonated sites of the interpolymer network. The distribution coefficients (K_d_, mL·g^−1^) were calculated according to Equation (2) to quantify the sorption preference. The calculated values were K_d_(Re) = 4.5 mL·g^−1^ and K_d_(Mo) = 2.0 mL·g^−1^, yielding a selectivity coefficient of β_Re/Mo_ = 2.25. These results demonstrate a moderate, but distinct, preference of the PMAA–P4VP system for Re(VII) oxyanions. This preference is consistent with the stronger electrostatic interactions between the protonated pyridyl nitrogen atoms and the monovalent ReO_4_^−^ ions, compared to the interaction with the divalent MoO_4_^2−^ species.

The selectivity pattern observed here supports the mechanism proposed in the pH-dependent study (Section 3.11), where the protonation of carboxyl and pyridyl groups enhances both electrostatic and donor–acceptor interactions. Furthermore, the β_Re/Mo_ ratio aligns with the FTIR observations (Section 3.14), confirming the involvement of both –COOH and –C_5_H_4_N groups in coordinating the oxyanions.

### 3.12. Desorption of Rhenium and Molybdenum Ions from PMAA–P4VP Interpolymer Systems

Desorption of Re(VII) and Mo(VI) ions was carried out using hydrochloric acid solutions of 4.0 M concentration at 25 ± 1 °C. The solid-to-liquid ratio was kept constant at 0.10 g per 100 mL of eluent. After equilibration for 48 h (identical to the sorption equilibration time), the desorbed concentration (Cd) was measured, and the desorption efficiency (D, %) was calculated according to Equation (12). Efficiency depended on polymer composition. The 2:4 system released the most significant amounts: 47.26 mg/L of Re and 17.38 mg/L of Mo. The 3:3 system also showed significant release (26.07 mg/L Re; 11.61 mg/L Mo), confirming good regeneration capacity. Systems rich in P4VP (1:5, 0:6) demonstrated moderate desorption, while PMAA-dominated systems (5:1, 6:0) released only small amounts.

**Figure 11:**
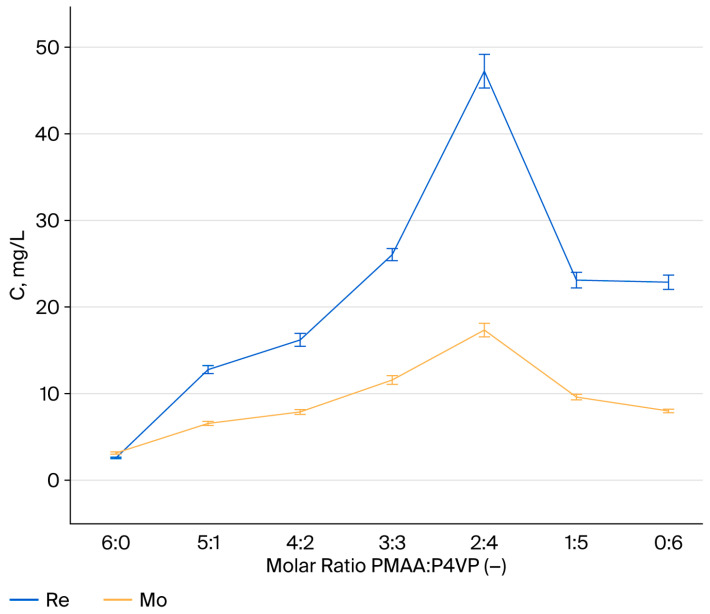
Desorption of rhenium (Re) and molybdenum MoO_4_^2−^ ions from PMAA–P4VP interpolymer systems with different molar ratios of components. Conditions: 4 M HCl as eluent; desorption time 24 h. Error bars represent the standard deviation (mean ± SD, *n* = 3), with SD ≤ 2%.

Figure 11 summarizes the comparative desorption behavior. The PMAA–P4VP system demonstrated high regeneration efficiency (≈90%) after three sorption-desorption cycles, confirming its potential for industrial-scale applications. The system’s performance under mild pH conditions (≈5.2) and its ability to retain stability and efficiency through multiple cycles indicate its practicality for the selective recovery of Re(VII) and Mo(VI) from hydrometallurgical effluents.

The developed interpolymer systems demonstrate higher or comparable adsorption capacities under mild conditions, highlighting their potential for selective oxyanion recovery.

### 3.13. FTIR Spectroscopic Study of the PMAA–P4VP Interpolymer System

**Figure 12:**
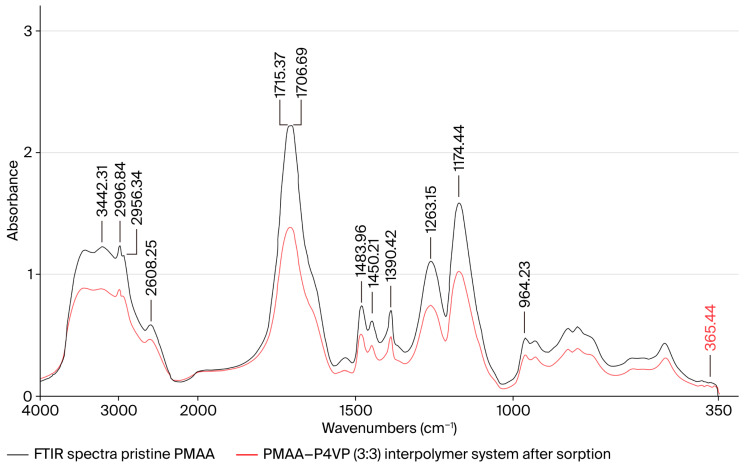
FTIR spectra of: pristine PMAA; PMAA–P4VP (3:3) interpolymer system after sorption of rhenium and molybdenum ions.

FTIR spectra of the PMAA–P4VP system were recorded before and after sorption of Re and Mo ions (Figure 12). In the spectrum of pristine PMAA, absorption in the 3300–2500 cm^−1^ range corresponds to νOH stretching of hydroxyl groups and bound water. Bands at 2994.91 and 2954.41 cm^−1^ are assigned to νC–H stretching of methyl and methylene groups [55,56]. The band at 2604.39 cm^−1^ reflects hydrogen bonding in carboxylic acid dimers. Strong signals at 1715.37 and 1706.69 cm^−1^ are due to νC=O of carboxyl groups [55]. Deformation modes of CH_3_ and CH_2_ groups appear at 1483.96, 1450.21, and 1390.42 cm^−1^, while νC–O stretching is observed at 1263.15 and 1174.44 cm^−1^ [57]. Out-of-plane OH vibrations occur at 964.23 cm^−1^ [58].

After sorption, the carbonyl bands decrease in intensity and shift slightly, indicating participation of carboxyl groups in metal coordination. Attenuation of the νOH region suggests disruption of hydrogen bonding upon complex formation. Weak additional features up to 367.44 cm^−1^ correspond to Me–O vibrations, confirming the binding of metal ions.

### 3.14. FTIR Spectroscopic Study of P4VP and PMAA–P4VP System After Metal Sorption

**Figure 13:**
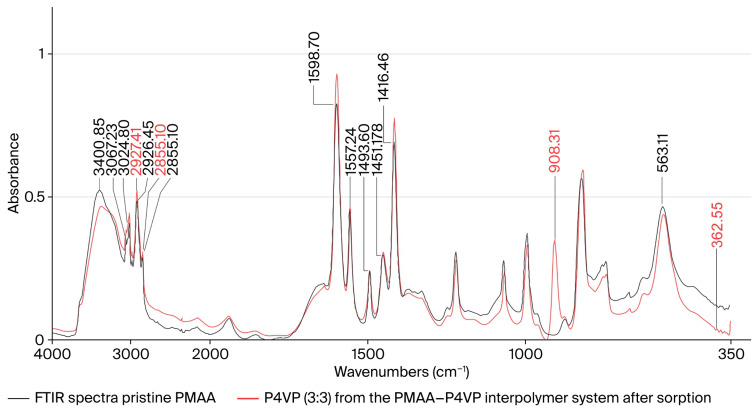
FTIR spectra: pristine P4VP; P4VP (3:3) from the PMAA–P4VP interpolymer system after sorption of rhenium and molybdenum ions.

The contribution of pyridine groups was examined by comparing the IR spectra of pure P4VP and P4VP after metal uptake within the PMAA–P4VP system (Figure 13). In the spectrum of unmodified P4VP, broad νOH bands are present at 3300–2500 cm^−1^ [55,56]. The band at 3024.80 cm^−1^ corresponds to aromatic νC–H stretching. Vibrations of νC=C and νC=N in pyridine rings appear in the 1600–1400 cm^−1^ region [55]. Stretching of methylene groups is observed at 2926.45 and 2855.10 cm^−1^, with deformation at 1451.17 cm^−1^ [55,56]. A band at 563.11 cm^−1^ is associated with conformational ordering of polymer chains [57]. Although direct IR evidence of pyridinium cation (N^+^) formation is limited, the observed spectral changes—particularly in νC=N and νOH regions—are consistent with protonation or alkylation of pyridine nitrogen during polymer modification and metal uptake.

After sorption, marked changes are observed: the νOH region is redistributed, νC=C and νC=N bands are preserved and intensified, and νCH_2_ vibrations (2927.41 and 2855.10 cm^−1^) increase in intensity. A new band at 908.31 cm^−1^ corresponds to the ν_3_(F_2_) vibration of [${ReO}_{4}^{-}$], while a signal at 362.55 cm^−1^ corresponds to the ν_4_(F_2_) vibration of [${MoO}_{4}^{2-}$] [56,57,58,59]. These features confirm the involvement of pyridine groups in complexation with Re and Mo ions and the formation of stable coordination structures.

As summarized in Table 3, the observed red shifts in C=O and C=N stretching vibrations (by 10–20 cm^−1^) indicate coordination-type interactions between the metal oxyanions and the protonated carboxyl and pyridyl groups in the interpolymer network. These findings confirm the cooperative binding of Re(VII) and Mo(VI) species through both electrostatic attraction and donor–acceptor coordination, consistent with the spectral features shown in Figure 12 and Figure 13. The red shift in the ν(C=O) band (≈25 cm^−1^) and attenuation of its intensity indicate that carboxyl groups of PMAA participate in coordination and ionic association with Re(VII) and Mo(VI) oxyanions. The pyridyl ν(C=N) band (≈1590 cm^−1^) shows a moderate downshift (11–12 cm^−1^), confirming donor–acceptor interaction with metal centers. Diagnostic absorptions at 908 cm^−1^ and 363 cm^−1^ correspond to the ν_3_(F_2_) and ν_4_(F_2_) modes of [ReO_4_^−^] and [MoO_4_^2−^], respectively, consistent with the formation of coordinated oxyanion complexes. The broad O–H stretching region (3300–2500 cm^−1^) becomes less intense and more diffuse, indicating disruption of hydrogen bonding and rearrangement of the hydration shell upon metal sorption.

### 3.15. Thermogravimetric (TGA) and Differential Scanning Calorimetry (DSC) Analysis of the PMAA–P4VP (3:3) Interpolymer System and Its Complexes with Rhenium and Molybdenum

**Figure 14:**
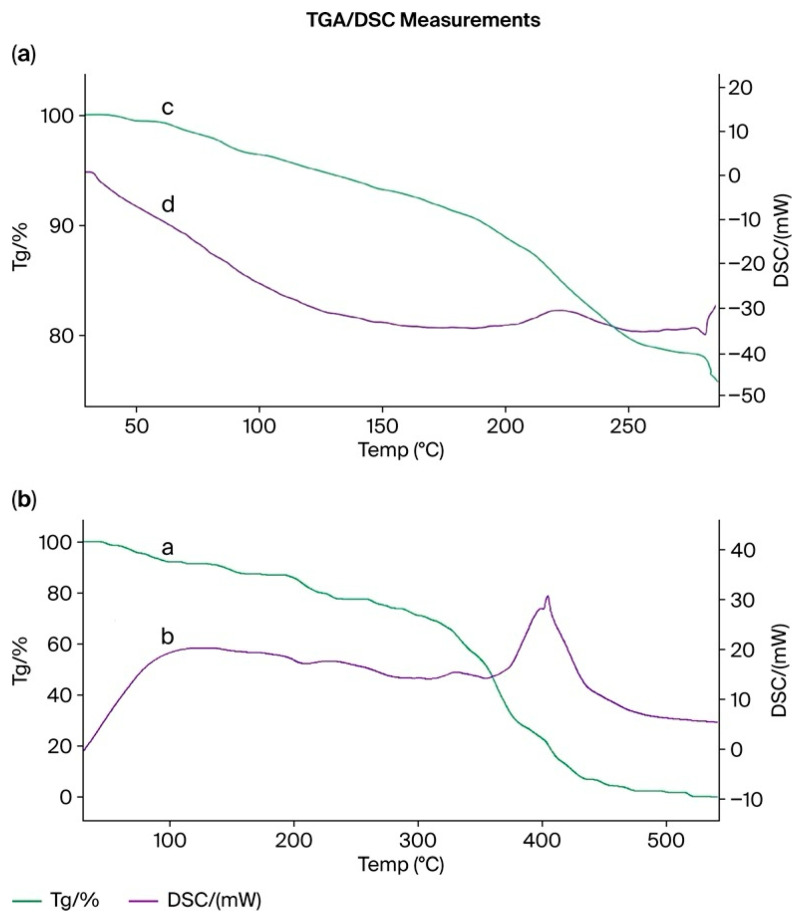
TGA and DSC curves for (**a**) pristine PMAA and (**b**) PMAA from the PMAA–P4VP (3:3) interpolymer system after sorption of rhenium and molybdenum ions. (**a**) Before sorption: curve c (green)—thermogravimetric (TGA) profile showing the mass loss (%) as a function of temperature; curve d (purple)—differential scanning calorimetry (DSC) curve showing the heat-flow changes (mW) during heating. (**b**) After sorption: curve a (green)—TGA profile after metal uptake; curve b (purple)—DSC curve showing the modified thermal response of the polymer complex.

TGA and DSC curves for (a) pristine PMAA and (b) PMAA from the PMAA–P4VP (3:3) interpolymer system after sorption of rhenium and molybdenum ions are shown in Figure 14. The DSC curve of the PMAA–P4VP interpolymer exhibits an endothermic peak near 110 °C, corresponding to moisture release and the glass transition of the polymer network, followed by an exothermic transition around 310 °C attributed to cross-link rearrangement and partial decomposition of carboxyl-containing fragments. After metal sorption, both transitions shift slightly toward higher temperatures, indicating enhanced structural stability of the interpolymer due to coordination bonding between the polymeric functional groups and the metal ions. Pure P4VP (Figure 14a) exhibits a minor mass loss below 120 °C due to moisture release and a major decomposition step between 200–300 °C, leaving ~37% residue. After sorption (Figure 14b), the PMAA–P4VP (3:3) system demonstrates delayed degradation onset, slower mass-loss rates, and altered DSC transitions, consistent with structural reinforcement through metal coordination. The slightly reduced residue (~32%) indicates partial modification of the polymer network, while overall thermal stability is maintained. These results suggest that coordination with Re(VII) and Mo(VI) reinforces the interpolymer network, improving its structural integrity at elevated temperatures.

**Figure 15:**
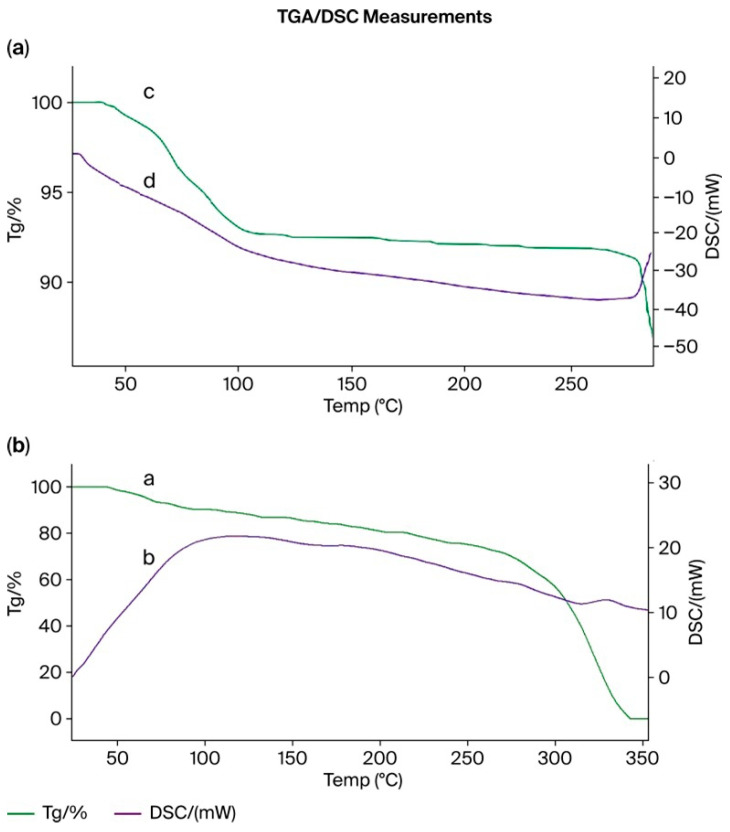
TGA and DSC curves for (**a**) pristine P4VP/ECH gel and (**b**) P4VP/ECH gel from the PMAA–P4VP (3:3) interpolymer system after sorption of rhenium and molybdenum ions. (**a**) Before sorption: curve c (green)—thermogravimetric (TGA) profile showing the mass loss (%) as a function of temperature. curve d (purple)—differential scanning calorimetry (DSC) curve showing the heat-flow variation (mW) with temperature. (**b**) After sorption: curve a (green)—TGA profile after metal ion sorption, illustrating a delayed onset of decomposition and improved thermal stability. Curve b (purple)—DSC curve demonstrating the thermal effects associated with complex formation and coordination of Re(VII) and Mo(VI) ions.

Pure P4VP (Figure 15a) exhibits mass loss below 120 °C (moisture) and major decomposition between 200–300 °C, with ~37% residue. After sorption (Figure 15b), the PMAA–P4VP (3:3) system demonstrates delayed degradation, slower mass-loss rates, and altered DSC transitions, consistent with structural reinforcement by metal coordination. The residual mass (~32%) is slightly lower, but the overall thermal stability remains high. These results indicate that interactions with Re and Mo enhance the structural organization and thermal resistance of the PMAA–P4VP network, supporting its suitability for applications under elevated thermal conditions.

These thermal effects are consistent with the FTIR results (Section 3.15), which revealed coordination of Re(VII) and Mo(VI) ions with carboxyl and pyridyl groups. The formation of such coordination bonds restricts segmental mobility of the polymer chains and stabilizes the interpolymer framework, leading to higher decomposition temperatures and modified endothermic–exothermic behavior observed in the DSC profiles.

### 3.16. Modeling of Sorption Isotherms: Linear and Nonlinear Approaches

**Figure 16:**
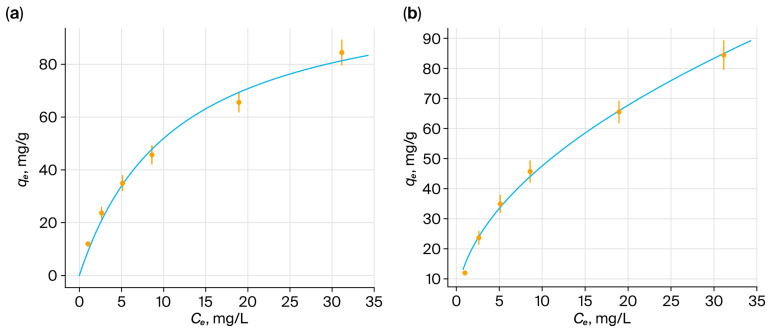
Nonlinear (**a**) Langmuir and (**b**) Freundlich adsorption isotherms for Re(VII) oxyanions sorbed onto PMAA–P4VP (2:4) interpolymer systems at 298 K. Equilibrium was established after 48 h of sorption, as confirmed by kinetic data (Section 3.17). Error bars represent the standard deviation (mean ± SD, *n* = 3), with SD ≤ 2%.

**Figure 17:**
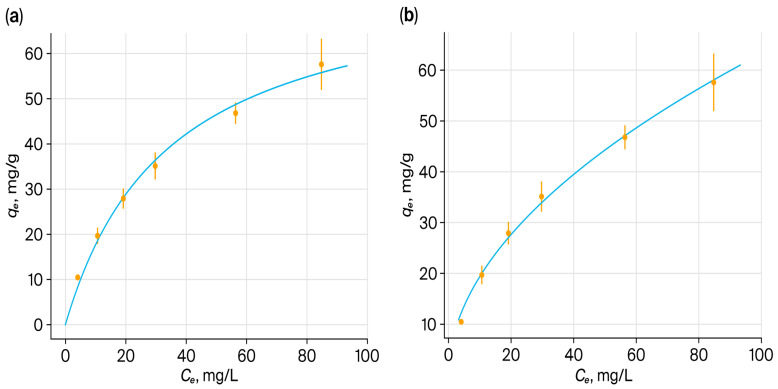
Nonlinear (**a**) Langmuir and (**b**) Freundlich adsorption isotherms for Mo(VI) oxyanions sorbed onto PMAA–P4VP (2:3) interpolymer systems at 298 K. Equilibrium conditions correspond to 48 h contact time verified through preliminary kinetics. Error bars represent the standard deviation (mean ± SD, *n* = 3), with SD ≤ 2%.

Equilibrium sorption of ReO_4_^−^ and MoO_4_^2−^ on PMAA–P4VP was analyzed using Langmuir and the Freundlich isotherm models in a nonlinear form. The Dubinin–Radushkevich model was excluded, as its assumptions are not suitable for hydrated, non-microporous hydrogels. For the Langmuir model, nonlinear fitting (Figure 16a) provided excellent correlations for rhenium (R^2^ > 0.999), yielding clearly defined q_max_ and b values. For molybdenum, nonlinear analysis gave R^2^ = 0.9913 (Figure 15a), confirming preferential binding at discrete adsorption sites. For the Freundlich model, nonlinear curves (Figure 16b and Figure 17b) adequately described the data, reflecting sorption on heterogeneous sites and possible multilayer adsorption. All isotherm data correspond to equilibrium conditions established after 48 h of sorption at 298 K. The equilibrium time was determined based on preliminary kinetic experiments (Section 3.17), in which the concentrations of Re(VII) and Mo(VI) oxyanions remained constant beyond 24–48 h, confirming the attainment of equilibrium.

Overall, the nonlinear analysis provided a statistically consistent description of the equilibrium data, minimizing distortions associated with linearized transformations. Although the Langmuir equation is traditionally based on the assumption of monolayer coverage over a uniform surface, its use here is primarily empirical, aimed at estimating the limiting sorption capacity (q_max_) and the apparent binding constant (b). In swollen interpolymer hydrogels, the sorption environment is heterogeneous; however, the Langmuir fit remains useful for comparative purposes and for describing the dominant coordination–electrostatic interaction sites within the matrix. In contrast, the Freundlich model reflects the intrinsic heterogeneity of polymer–metal binding, and the combined interpretation of both models provides a realistic representation of the sorption mechanism. These results indicate that the PMAA–P4VP system combines homogeneous and heterogeneous sorption features, ensuring effective uptake of Re(VII) and Mo(VI) (Table 4).

**Figure 18:**
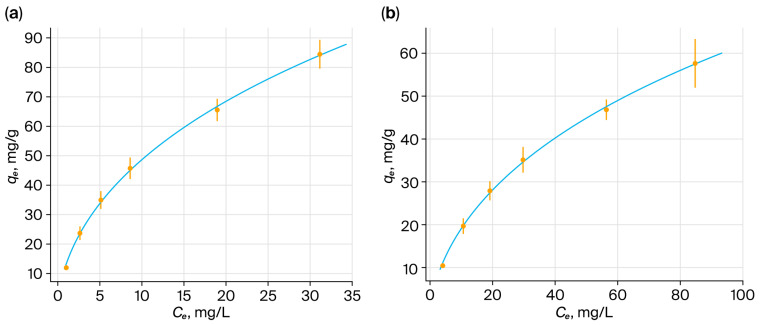
Nonlinear Dubinin–Radushkevich (**a**) Re(VII) and (**b**) Mo(VI) adsorption isotherms for Mo(VI) oxyanions sorbed onto PMAA–P4VP (2:3) interpolymer systems at 298 K. Equilibrium conditions correspond to 48 h contact time verified through preliminary kinetics. Error bars represent the standard deviation (mean ± SD, *n* = 3), with SD ≤ 2%.

Isotherms (Figure 18) illustrate the sorption capacity of the PMAK-P4VP (2:3) interpolymer system for rhenium and molybdenum oxyanions. In both cases, the isotherms obey the nonlinear Dubinin–Radushkevich model, reflecting the complex interaction of the polymer matrix with metal ions. The system’s ability to sorb rhenium (a) and molybdenum (b) ions demonstrates significant differences in sorption behavior, potentially related to the structural features of the interpolymer and the nature of the oxyanions.

### 3.17. Kinetics (Preliminary Analysis)

To complement the equilibrium analysis, a preliminary evaluation of sorption kinetics was performed for the representative PMAA:P4VP systems 2:4 and 3:3. Experimental data were fitted using pseudo-first-order (PFO), pseudo-second-order (PSO), and intraparticle diffusion (Weber–Morris) models. The obtained parameters are summarized in Table 5. For the rhenium system (ReO_4_^−^), the 2:4 composition exhibited moderate agreement with the PFO and PSO models (R^2^ = 0.36–0.47), while the Weber–Morris model provided a better fit (R^2^ = 0.81), indicating the significance of diffusion-controlled processes within the polymer network. For molybdenum (MoO_4_^2−^), the PSO model yielded a slightly higher determination coefficient (R^2^ ≈ 0.80) for the 2:4 system, indicating a chemisorption contribution. In contrast, the 3:3 composition followed mixed kinetics, with similar fits across all three models (R^2^ ≈ 0.96). The intraparticle diffusion model revealed the importance of boundary layer thickness, which relates to the concentration gradient between the solid surface and the bulk solution. In this model, when C = 0, this means that the sorbate concentration in the boundary layer has reached equilibrium with the surrounding solution, and there is no further mass transfer at the boundary. Thickness plays a crucial role in controlling the sorption rate, as a thicker boundary layer can slow down the diffusion process, limiting the movement of ions into the polymer matrix. Furthermore, boundary layer thickness, in combination with intraparticle diffusion, affects the overall sorption kinetics in the PMAA–P4VP system. With a thinner boundary layer, ions can diffuse more efficiently into the polymer network, resulting in higher sorption rates and overall efficiency. Understanding this interaction helps optimize sorption conditions and improve the design of interpolymer systems for more efficient metal ion extraction.

Kinetic parameters obtained from the pseudo-first-order (PFO), pseudo-second-order (PSO), and intraparticle diffusion (Weber–Morris) models for ${ReO}_{4}^{-}$ and ${MoO}_{4}^{2-}$ sorption on PMAA–P4VP interpolymer systems. The PSO model provides the best fit (R^2^ > 0.96), indicating chemisorption as the rate-controlling step, while the Weber–Morris model reveals a mixed surface and intraparticle diffusion mechanism. Experimental conditions: C_0_ = 110 mg·L^−1^; V = 50 mL; m = 0.10 g; T = 25 ± 1 °C; pH = 5.2.

**Figure 19:**
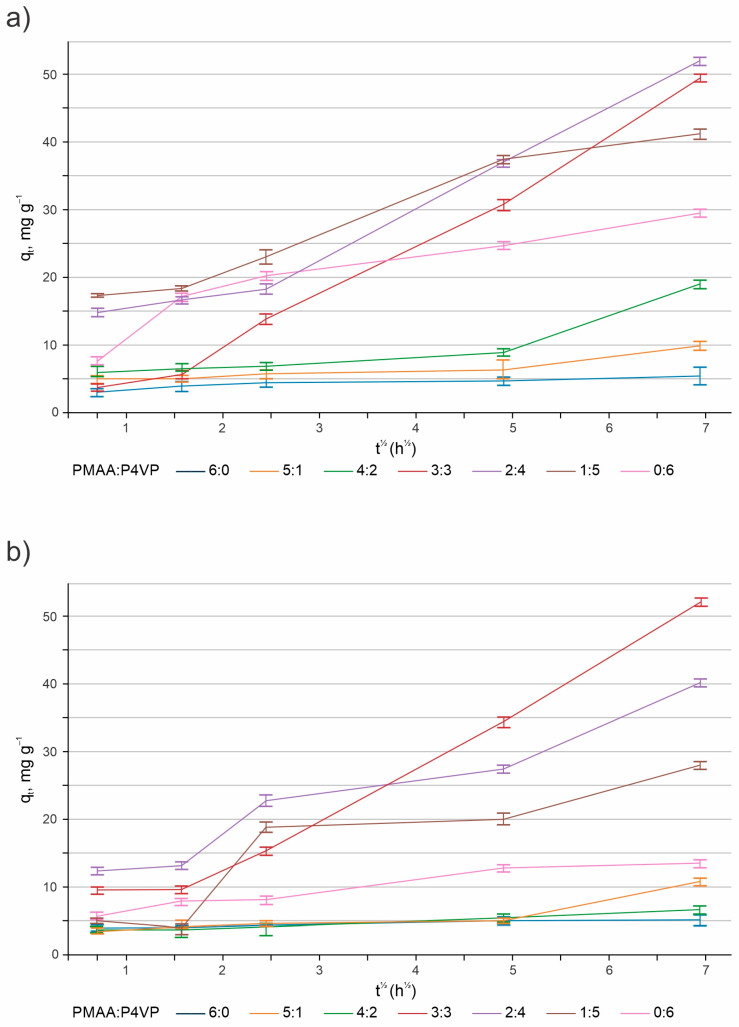
Weber–Morris intraparticle diffusion plots for the sorption of (**a**) ReO_4_^−^ and (**b**) MoO_4_^2−^ ions on PMAA–P4VP interpolymer systems at various molar ratios (6:0, 5:1, 4:2, 3:3, 2:4, 1:5, and 0:6). Error bars represent the standard deviation (mean ± SD, *n* = 3), with SD ≤ 2%.

The Weber–Morris intraparticle diffusion model (Equation (6)) was used to analyze the data. A multistep sorption process is indicated by the multiple linear regions for ReO_4_^−^ (Figure 19a) and MoO_4_^2−^ (Figure 19b) in the plots of *q*_*t*_ versus *t* ^1/2^. A slower linear region linked to intraparticle diffusion follows the first segment, which is associated with a faster film diffusion process. The saturation of available sorption sites is responsible for the observation of a third region that approaches equilibrium in certain cases, namely for ReO_4_^−^ in the PMAA:P4VP = 3:3 system and MoO_4_^2−^ in the 2:4 system [61,62,63]. Consistent with previous research, a non-zero intercept (C) signifies the existence of a boundary layer effect [64]. Film diffusion is present because the plot does not cross the origin, and the initial adsorbate concentration is nonzero [65,66,67,68,69].

For ReO_4_^−^:

Because of the quick electrostatic attraction to the pyridinium centers, interpolymer systems with a predominance of P4VP (1:5 and 0:6) showed a high K_i,1_ value. Nevertheless, a brief stabilization phase with decreased K_i,2_ values ensued. Systems with a balanced composition (2:4 and 3:3) showed the highest overall sorption capacity, stable intraparticle diffusion (high K_i,2_), and moderate initial sorption. There were three different kinetic stages in the 3:3 system.

For MoO_4_^2−^:

In the second region, the 3:3 and 2:4 systems showed clear linearity and slope, suggesting effective intraparticle diffusion and advantageous coordination with both the –COOH and –C_5_H_4_N functional groups. P4VP-dominated systems exhibited a slower diffusion phase in the second region (lower K_i,2_) but a fast initial sorption (high K_i,1_). A third kinetic region was seen in the 2:4 system, indicating prolonged diffusion into the deeper polymer domains.

### 3.18. Surface Morphology

**Figure 20:**
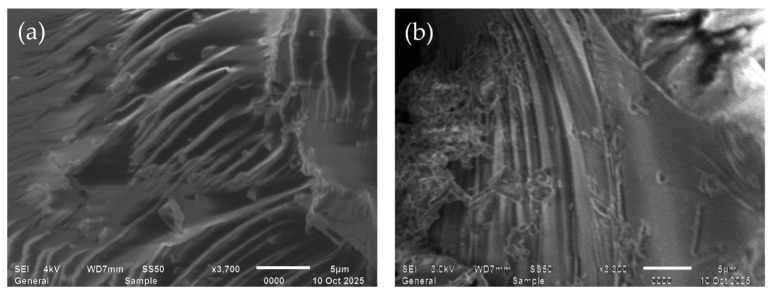
SEM images of PMAA hydrogel: (**a**) pristine sample, (**b**) after Re(VII) and Mo(VI) sorption.

**Figure 21:**
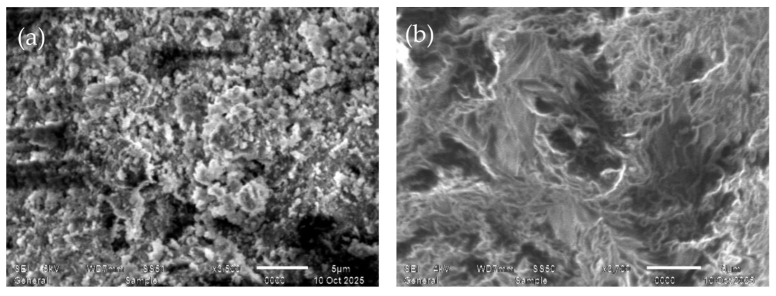
SEM images of P4VP hydrogel: (**a**) pristine sample, (**b**) after Re(VII) and Mo(VI) sorption.

The surface morphology of the pristine and metal-loaded PMAA and P4VP hydrogels was investigated using scanning electron microscopy. Imaging was performed at accelerating voltages of 5–10 kV under low-vacuum conditions (<9 × 10^−3^ Pa) to minimize charging and beam-induced degradation. Samples were mounted on double-sided conductive tape and coated with a thin conductive layer prior to imaging. Micrographs were acquired using low-frequency scanning to ensure high-quality visualization. Magnifications ranged from 3× to 1,000,000×, with a field of view of 7.7 mm at a 10 mm working distance and 24 mm at a 30 mm. The system offered energy resolution <129 eV (Mn K_β_), detector area 30 mm^2^, and maximum count rates exceeding 10^6^ counts/s. Elemental mapping (EDS) was available for qualitative and quantitative analysis. SEM images (Figure 20 and Figure 21) show that the pristine PMAA hydrogel exhibits a relatively smooth layered structure with well-defined lamellae, characteristic of poly(methacrylic acid) networks.

The pristine PMAA exhibits a relatively smooth layered surface with parallel lamellae characteristic of poly(methacrylic acid) gels. After sorption, the surface becomes more heterogeneous and compact, indicating the formation of Re–O–C coordination sites and partial structural densification of the network.

The initial P4VP surface shows a granular and porous morphology, while after sorption, the polymer becomes denser and more folded, reflecting interactions between pyridyl nitrogen atoms and ReO_4_^−^ ions and a decrease in pore size due to coordination and electrostatic binding.

### 3.19. Comparison with Literature Data

The performance of the PMAA–P4VP system aligns with previous studies reporting synergistic effects between acidic and basic polymeric groups [13]. Compositions with ratios of 2:4 and 3:3 achieved the highest sorption efficiencies, in agreement with the findings of Wang et al. [70], who reported that cooperative ionization and conformational flexibility enhance metal binding. Similar behavior has been observed for mesoporous sorbents [22], magnetically modified polymers [21], and ion-exchange resins [18,19]. In Table 2 the sorption capacities and pH optima from relevant studies are listed, showing a comparison with the PMAA–P4VP system. To provide better context for the comparison of sorption capacities, additional references have been included throughout the Results and Discussion sections. These references highlight key studies that report similar sorption behavior for Re(VII) and Mo(VI) ions, providing a more comprehensive analysis of the PMAA–P4VP system.

The experimentally obtained equilibrium adsorption capacities (q_e_, mg g^−1^) were calculated from equilibrium concentrations and used for quantitative comparison with literature data (Table 2). The developed PMAA–P4VP systems exhibited q_e_ values of 48.6 mg g^−1^ for Re(VII) and 42.7 mg g^−1^ for Mo(VI), which are comparable to or higher than those reported for many polymer-based sorbents. For instance, a magnetic Fe_3_O_4_–P4VP composite reached 41.7 mg g^−1^ for Re(VII) [14], while chitosan-based sorbents exhibited 124 mg g^−1^ for Mo(VI) [52]. The Mo(VI) ion-imprinted polymer reported by Fallah et al. [51] showed 126 mg g^−1^ capacity at pH 3–4, and the magnetic Cr-ferrite nanocomposite synthesized by Gamal et al. [50] achieved about 27 mg g^−1^ under similar conditions. Commercial anion-exchange resins Purolite A170 and Dowex 21 K demonstrated 166.7 and 142.9 mg g^−1^ for Re(VII) adsorption [48], whereas functionalized alumina and carbon materials showed significantly lower capacities—31 mg g^−1^ for γ-Al_2_O_3_ [71] and 16.5 mg g^−1^ for activated carbon [72].

Comparable inorganic adsorbents such as NH_4_HCO_3_-modified Al_2_O_3_ exhibited only 1.94 mg g^−1^ at strongly acidic pH 2–3 [47], highlighting the superior affinity of polymeric systems. Magnetic or oxide-based composites—such as tungstate-modified Fe_3_O_4_ (182 mg g^−1^) [73] and titanate–FeS nanocomposites (226 mg g^−1^) [74]—achieve higher absolute capacities but rely on highly acidic or redox-active environments, limiting their recyclability.

In contrast, the PMAA–P4VP interpolymer system operates efficiently under mild aqueous conditions (pH ≈ 5.2, 298 K) without added electrolytes or extreme pretreatment. Its balanced acid–base composition enables cooperative utilization of carboxyl (–COOH) and pyridyl (–C_5_H_4_N) groups, ensuring both high affinity and ≈90% regenerability after three cycles. This combination of moderate but stable capacity, chemical robustness, and near-neutral working pH positions PMAA–P4VP hydrogels as environmentally benign and practically competitive materials for the selective recovery of Re(VII) and Mo(VI) oxyanions from hydrometallurgical effluents.

### 3.20. Limitations and Future Work

The present study was conducted using single-ion model systems containing Re(VII) and Mo(VI) oxyanions to establish the fundamental physicochemical principles governing their sorption by the PMAA–P4VP interpolymer network. While such conditions ensure reproducibility and allow precise evaluation of composition and pH effects, they do not fully represent the complexity of real hydrometallurgical environments. In industrial leachates and process streams, target ions coexist with various competing species that may influence selectivity and binding efficiency. Future work will therefore focus on testing the PMAA–P4VP system in controlled multicomponent synthetic solutions containing sulfate (SO_4_^2−^), chloride (Cl^−^), ferric (Fe^3+^), and copper (Cu^2+^) ions. These studies will help assess competitive sorption mechanisms, determine distribution and selectivity coefficients, and validate the applicability of the developed system for the selective recovery of rhenium and molybdenum from complex industrial solutions.

## 4. Conclusions

The interpolymer system composed of poly(methacrylic acid) (PMAA) and poly(4-vinylpyridine) (P4VP) exhibits pronounced cooperative effects between acidic (–COOH) and basic (–C_5_H_4_N) functional groups, which account for its strong affinity toward Re(VII) and Mo(VI) oxyanions. The compositions with PMAA:P4VP ratios of 2:4 and 3:3 showed the highest sorption performance, reaching recovery degrees of 91.7% for Re(VII) and 94.6% for Mo(VI) at pH ≈ 5.2 and 298 K after 48 h of contact. Spectroscopic data confirmed the participation of both functional groups in metal-ion coordination. In the IR spectra, the ν(C=O) stretching band shifted from 1715 cm^−1^ to 1685 cm^−1^, and the ν(C=N) band from 1594 cm^−1^ to 1582 cm^−1^. The appearance of new absorption peaks near 908 cm^−1^ (Re–O) and 863 cm^−1^ (Mo–O) indicates the formation of perrhenate and molybdate complexes. Thermogravimetric analysis revealed an increase in the decomposition temperature by approximately 30 °C after sorption, reflecting the enhanced rigidity and thermal stability of the polymer framework. The sorbent preserved its morphology and structural integrity after three successive sorption–desorption cycles, maintaining about 90% of its initial capacity. Scanning electron microscopy revealed a porous and heterogeneous surface that favors ion transport through the polymer matrix. Kinetic modeling showed that the pseudo-second-order (PSO) model accurately describes the experimental results (R^2^ > 0.96). The rate of sorption is influenced by both chemisorption and intraparticle diffusion processes. The sorption of ReO_4_^−^ and MoO_4_^2−^ on PMAA–P4VP interpolymer systems occurs through multiple diffusion stages involving both film and intraparticle mechanisms, according to the kinetic analysis based on the Weber–Morris intraparticle diffusion model. The most effective overall sorption performance was shown by the balanced compositions (2:4 and 3:3), which combined high equilibrium capacity, prolonged intraparticle diffusion, and quick initial uptake. Among the equilibrium models examined, the nonlinear Langmuir isotherm provided the best fit (R^2^ ≈ 0.99), with maximum adsorption capacities of 48.6 mg g^−1^ for Re(VII) and 42.7 mg g^−1^ for Mo(VI). The Dubinin–Radushkevich analysis suggests that ion-exchange and coordination mechanisms dominate over purely physical adsorption, with mean adsorption energies of 8–9 kJ mol^−1^.

In conclusion, the PMAA/P4VP interpolymer system offers a novel approach as a structurally stable, regenerable, and selective sorbent for oxyanion recovery. Its well-balanced acid-base composition, effective performance under mild pH conditions, and strong resilience during multiple regeneration cycles position it as a promising candidate for sustainable hydrometallurgical processes, particularly for the recovery of rhenium and molybdenum from complex industrial effluents in Kazakhstan’s metallurgical sector. Additionally, the scalability of the PMAA–P4VP system makes it suitable for large-scale applications. The system can be synthesized using conventional polymerization techniques and demonstrates efficient regeneration, with approximately 90% recovery after three cycles. This makes it a sustainable and cost-effective alternative to conventional ion-exchange resins in hydrometallurgical recovery processes. Moreover, its environmentally friendly synthesis using water-soluble monomers contributes to reduced chemical waste, making PMAA–P4VP an attractive option for sustainable metal recovery.

## Figures and Tables

**Table 1:** Statistical parameters and adsorption constants from nonlinear isotherm modeling.

Model	R^2^	RMSE	χ^2^	AIC	q_m_ (mg·g^−1^)	Kₗ (L·mg^−1^)
Langmuir	0.998	0.012	0.003	24.5	48.6	0.045
Freundlich	0.976	0.034	0.009	31.2	—	—

**Table 2:** Adsorption capacities of Re(VII) and Mo(VI) on various polymeric and inorganic sorbents.

Sorbent System	Target Ion	q_e_/q_max_ (mg g^−1^)	pH/Conditions	Equilibrium Time (min)	Model/Mechanism	Reference
PMAA–P4VP (2:4)	Re(VII)	48.6	pH 5.2, 298 K	2880	PSO, Langmuir (monolayer)	This work
PMAA–P4VP (3:3)	Mo(VI)	42.7	pH 5.2, 298 K	2880	PSO, Langmuir (monolayer)	This work
NH_4_HCO_3_–modified nano-Al_2_O_3_	Re(VII)	1.94	pH 2–3, 298 K	120	PSO, Langmuir (monolayer)	[47]
Purolite A170 resin (weak-base)	Re(VII)	166.7	pH 5.0, 298 K	60	PSO, Freundlich + D-R, ion Exchange	[48]
Dowex 21 K resin (strong-base)	Re(VII)	142.9	pH 5.0, 298 K	60	PSO, Freundlich + D-R	[48]
γ-Al_2_O_3_	Mo(VI)	31.0	pH 4.0	120	PFO	[49]
Magnetic Cr-ferrite	Mo(VI)	26.8	pH 4.0	180	PSO	[50]
Ion-imprinted polymer	Mo(VI)	126.1	pH 3–4	10	Langmuir (monolayer)	[51]
Chitosan sorbent	Mo(VI)	124.3	pH 5.0	15	PSO (chemisorption)	[52]
Modified water-treatment residue	Mo(VI)	39.5	pH 6.0	—	Freundlich (heterogeneous surface)	[53]
Activated carbon	Mo(VI)	16.5	pH 4.0	30	PFO (physical adsorption)	[54]

**Table 3:** FTIR band positions of PMAA–P4VP before and after sorption of Re(VII) and Mo(VI) (resolution ± 2 cm^−1^).

Functional Group	Assignment	ν (Before), cm^−1^ (± 2)	ν (After Re(VII)), cm^−1^	Δν (cm^−1^)	ν (After Mo(VI)), cm^−1^	Δν (cm^−1^)	ReferenceBandrange (lit.), cm^−1^	Interpretation
–COOH/–COO^−^	ν(C=O) stretch (PMAA)	1715/1707	1685	−25	1688	−22	1695–1715 [55,56]	Coordination via carboxyl oxygen
–C=N (pyridyl)	ν(C=N) stretch (P4VP)	~1594	~1582	−12	~1583	−11	1585–1605 [55,56]	Donor–acceptor interaction with metal center
–C–O (acidic)	ν(C–O) stretch	1263/1174	~1190	−10 −15	~1195	−10	1170–1270 [57]	Deprotonation and participation in binding
Re–O	ν_3_(F_2_) ReO_4_^−^	—	908	+908	—	—	910–930 [56,57,58]	Formation of perrhenate complex
Mo–O	ν_4_(F_2_) MoO_4_^2−^	—	—	—	363	+363	850–900 [57,58,59]	Formation of molybdate complex
O–H (H-bonded)	ν(O–H) broad	3300–2500	broad	—	broad	—	2500–3500 [60]	Hydrogen-bond reorganization

Notes: ν—vibration frequency; Δν—shift relative to the initial spectrum. Negative Δν values indicate red shifts associated with coordination or hydrogen-bond weakening.

**Table 4:** Equilibrium and model parameters for the sorption of Re(VII) and Mo(VI) oxyanions on PMAA–P4VP interpolymer systems (nonlinear Langmuir, Freundlich, Dubinin–Radushkevich fitting at 298 K).

Ion	System (PMAA:P4VP)	q_e_ (48 h), mg·g^−1^	q_max_ (Langmuir), mg·g^−1^	b (L·mg^−1^)	Kd, mL·g^−1^	β_Re/Mo_	R^2^ (Langmuir)	R^2^ (Freundlich)	KDR (mol^2^∙kJ^−2^)	E (kJ∙mol^−1^)	R^2^ (Dubinin–Radushkevich)
Re(VII)	2:4	45.8 ± 1.3	48.6 ± 1.1	0.052	4450	—	0.999	0.982	1.2 × 10^−6^	20.4	0.961
Re(VII)	3:3	41.5 ± 1.1	43.2 ± 0.9	0.047	4170	—	0.993	0.978	1.4 × 10^−6^	18,9	0.957
Mo(VI)	2:4	38.1 ± 1.4	42.7 ± 1.0	0.039	3890	1.15	0.991	0.987	1.6 × 10^−6^	17.7	0.948
Mo(VI)	3:3	36.3 ± 1.2	40.2 ± 0.9	0.035	3560	1.17	0.985	0.982	1.9 × 10^−6^	16.2	0.942

Notes: Experimental conditions: C_0_ = 10–250 mg·L^−1^; V = 50 mL; m = 0.06 g; T = 25 ± 1 °C; pH = 5.2; equilibrium time = 48 h.

**Table 5:** Kinetic parameters obtained from pseudo-first-order (PFO), pseudo-second-order (PSO), and intraparticle diffusion (Weber–Morris) models for ReO_4_^−^ and MoO_4_^2−^ sorption on PMAA–P4VP interpolymer systems.

	System (PMAA:P4VP)	PFO Model			PSO Model			Intraparticle Diffusion (Weber–Morris)		
Ion		q_e_ (mg·g^−1^)	k_1_ (h^−1^)	R^2^	q_e_ (mg·g^−1^)	k_2_ (g·mg^−1^·h^−1^)	R^2^	k_id_ (mg∙g^−1^∙h^−1/2^)	C (mg∙g^−1^)	R^2^
*ReO*_4_^−^	2:4	51.180	0.0472	0.365	45.830	3.36 × 10^−3^	0.469	5.100	8.380	0.807
*ReO*_4_^−^	3:3	48.720	0.0545	0.849	44.250	2.95 × 10^−3^	0.849	6.170	−2.180	0.817
*MoO*_4_^2−^	2:4	34.180	0.197	0.725	38.650	6.24 × 10^−3^	0.797	4.450	8.360	0.958
*MoO*_4_^2−^	3:3	54.900	0.0614	0.962	73.180	7.28 × 10^−4^	0.962	7.740	−0.022	0.964

Notes: q_e_— equilibrium sorption capacity; k_1_ and k_2_— rate constants of the pseudo-first-order and pseudo-second-order models, respectively; k_id_— intraparticle diffusion rate constant; C—boundary layer thickness parameter.

## Data Availability

All data supporting the findings of this study are included within the article.

## Appendix A. Analysis and Comparison of Water Purification Strategies

**Table A1:** Evaluation of Various Water Treatment Techniques.

Method	Principle of Action	Advantages	Limitations	References
Membrane technologies (MF, UF, NF, RO)	Physical separation based on pore size and pressure	High efficiency, modularity	Membrane fouling, high cost	[19,20]
Chemical cleaning of membranes	Dissolution of contaminants using acids, alkalis, and oxidants	Rapid restoration of performance	Possible membrane damage, chemical waste	[19]
Photocatalytic treatment	Generation of reactive radicals (OH) under light	Effective against organic pollutants	Requires light and a photocatalyst, a slow process	[15]
Electrochemical treatment	Electric field destruction of pollutants or stimulation of reactions	No reagents, targeted effect	High energy consumption, complex equipment	[21]
Biological cleaning	Use of enzymes or bacteria to degrade contaminants	Environmentally friendly, mild conditions	Long reaction time, sensitivity to environmental conditions	[21]
Sorption (adsorption/absorption)	Capture of pollutants on the surface or within the sorbent structure	Simplicity, availability, and high selectivity	Sorbent saturation, need for regeneration	[22]
